# Signatures of Selection for Environmental Adaptation and Zebu × Taurine Hybrid Fitness in East African Shorthorn Zebu

**DOI:** 10.3389/fgene.2017.00068

**Published:** 2017-06-08

**Authors:** Hussain Bahbahani, Abdulfatai Tijjani, Christopher Mukasa, David Wragg, Faisal Almathen, Oyekanmi Nash, Gerald N. Akpa, Mary Mbole-Kariuki, Sunir Malla, Mark Woolhouse, Tad Sonstegard, Curtis Van Tassell, Martin Blythe, Heather Huson, Olivier Hanotte

**Affiliations:** ^1^Department of Biological Sciences, Faculty of Science, Kuwait UniversityKuwait, Kuwait; ^2^School of Life Sciences, University of NottinghamNottingham, United Kingdom; ^3^Centre for Genomics Research and Innovation, National Biotechnology Development AgencyAbuja, Nigeria; ^4^National Animal Genetic Resource Centre and Data BankEntebbe, Uganda; ^5^Centre for Tropical Livestock Genetics and Health, Roslin InstituteEdinburgh, United Kingdom; ^6^Department of Veterinary Public Health and Animal Husbandry, College of Veterinary Medicine, King Faisal UniversityAl-Hasa, Saudi Arabia; ^7^Department of Animal Science, Ahmadu Bello UniversityZaria, Nigeria; ^8^Deep Seq Department, University of NottinghamNottingham, United Kingdom; ^9^Ashworth Laboratories, Centre for Immunity, Infection and Evolution, University of EdinburghEdinburgh, United Kingdom; ^10^Recombinetics, Inc.St. Paul, MN, United States; ^11^Animal Genomics and Improvement Laboratory, United States Department of Agriculture, Agricultural Research ServiceBeltsville, MD, United States; ^12^International Livestock Research Institute (ILRI)Addis Ababa, Ethiopia

**Keywords:** African cattle, positive selection, environmental adaptation, hybrid fitness, meta-analysis of selection signals

## Abstract

The East African Shorthorn Zebu (EASZ) cattle are ancient hybrid between Asian zebu × African taurine cattle preferred by local farmers due to their adaptability to the African environment. The genetic controls of these adaptabilities are not clearly understood yet. Here, we genotyped 92 EASZ samples from Kenya (KEASZ) with more than 770,000 SNPs and sequenced the genome of a pool of 10 KEASZ. We observe an even admixed autosomal zebu × taurine genomic structure in the population. A total of 101 and 165 candidate regions of positive selection, based on genome-wide SNP analyses (*meta-SS, Rsb, iHS*, and Δ*AF*) and pooled heterozygosity (*Hp*) full genome sequence analysis, are identified, in which 35 regions are shared between them. A total of 142 functional variants, one novel, have been detected within these regions, in which 30 and 26 were classified as of zebu and African taurine origins, respectively. High density genome-wide SNP analysis of zebu × taurine admixed cattle populations from Uganda and Nigeria show that 25 of these regions are shared between KEASZ and Uganda cattle, and seven regions are shared across the KEASZ, Uganda, and Nigeria cattle. The identification of common candidate regions allows us to fine map 18 regions. These regions intersect with genes and QTL associated with reproduction and environmental stress (e.g., immunity and heat stress) suggesting that the genome of the zebu × taurine admixed cattle has been uniquely selected to maximize hybrid fitness both in terms of reproduction and survivability.

## Introduction

Domestic cattle are classified, based on phenotypes, into humpless taurine (*Bos taurus*) and humped zebu (*B. indicus*) cattle. They originated from different auroch ancestral populations *B. primigenius primigenius* and *B. p. namadicus* at two separate domestication centers; the Near East and the Indus Valley—Northern part of the Indian subcontinent, respectively (Epstein, 1971; Loftus et al., 1994; Troy et al., 2001; Chen et al., 2010). These two cattle types are considered as distinct species or subspecies with mitochondrial DNA (Loftus et al., 1994) and microsatellite (MacHugh et al., 1997) analyses suggesting a common ancestry about 200,000 and 700,000 years ago, respectively.

Cattle populations of zebu × African taurine ancestries are common in Africa and in particularly on the eastern part of Africa, Southern Africa, and along the Sahelian belt. According to their phenotypes, they are either referred to as African zebu, e.g., East African Shorthorn Zebu (EASZ) in Kenya, Adamawa Gudali in Nigeria, and Karamojong zebu in Uganda, African sanga, e.g., Ankole in Uganda, or African zenga e.g., Nganda in Uganda (Epstein, 1971; Rege, 1999; Rege et al., 2001). These cattle originated from the earlier migration of *B. taurus* and *B. indicus* to the continent from their putative centers of domestication followed by subsequent hybridization between them (Loftus et al., 1994; Chen et al., 2010; Gifford-Gonzalez and Hanotte, 2011). An African auroch influence in their genome has been postulated (Decker et al., 2014) but this remains until now speculative. The cattle colonization of the African continent started first with the arrival of taurine cattle, ~7,000 years ago (Gifford-Gonzalez and Hanotte, 2011), followed by the zebu type in two waves, ~4,000 and ~1,300 years ago, according to pictorial, archeological and genetic evidences (Epstein, 1971; Hanotte et al., 2002). The later was through the Horn of Africa and it was likely linked to the development of the Swahili civilization (Hanotte et al., 2002). Zebu × taurine hybrids are therefore of ancient origin on the African continent. Given the sole presence of the taurine type mtDNA in African cattle, a male-mediated introgression of zebu cattle to the native African taurine has been proposed (Loftus et al., 1994; Bradley et al., 1996). Microsatellite analysis (Hanotte et al., 2002) and more recently genome-wide single nucleotide polymorphism (SNP) analyses (Decker et al., 2014) show that the indicine ancestry peaks in the Horn of Africa gradually declining toward the western and the southern parts of the continent.

The small EASZ is the main type of African zebu cattle populating East Africa (Rege et al., 2001). As for other indigenous cattle populations, EASZ are more preferred by the local farmers over the pure exotic highly productive taurine breeds due to their superior adaptability to their local environment, which is characterized by a warm climate (20–23°C), high humidity (60–80%) and high pathogenes challenges (e.g., *Theileria parva, Ehrlichia ruminantium*, and *Haemonchus placei*; de Clare Bronsvoort et al., 2013). These cattle show a degree of resistance to *Rhipicephalus appendiculatus* tick, the vector of East Coast Fever (ECF) protozoan parasite *T. parva* (Latif et al., 1991; Latif and Pegram, 1992), as well as tolerance to poor forage and water scarcity (Western and Finch, 1986). A mortality rate of ~16% has been observed mainly attributed to ECF, haemonchosis, and heartwater in an EASZ population of western Kenya under the traditional management system (de Clare Bronsvoort et al., 2013; Thumbi et al., 2014). The genetic structure of this cattle population has recently been investigated by Mbole-Kariuki et al. (2014) using mid-density genome-wide SNP data. This study showed that the EASZ is a stabilized admixed population of zebu and African taurine ancestries, with an average genome proportions of 0.84 ± 0.009 and 0.16 ± 0.009, respectively. However, some of the EASZ animal showed recent European taurine introgression likely following artificial insemination program aiming to improve indigenous cattle productivity (Mbole-Kariuki et al., 2014). This European introgression has been linked to increased vulnerability of EASZ to infectious diseases (Murray et al., 2013).

The genomes of several livestock species have now been intensively explored for signatures of positive selection, e.g., chicken (Rubin et al., 2010; Zhang et al., 2012), pigs (Rubin et al., 2012), sheep (Kijas et al., 2012), and cattle (Gautier et al., 2009; Gautier and Naves, 2011; Kemper et al., 2014; Bahbahani et al., 2015). In cattle, the main genomic tool used for this purpose is the commercially available genome-wide SNP chip (e.g., Illumina BovineSNP50 BeadChip and Illumina BovineHD BeadChip). Although the lower-density SNP chip has been widely used to detect signatures of selection on the genome of tropically adapted cattle (Gautier et al., 2009; Gautier and Naves, 2011; Flori et al., 2012, 2014) and commercial dairy and beef breeds (Flori et al., 2009; Qanbari et al., 2011; Khayatzadeh et al., 2016), it has been associated with two main drawbacks; (i) the limited coverage of the bovine genome with an average markers gap of 49.4 kb, (ii) the SNP ascertainment bias toward European taurine breeds (Matukumalli et al., 2009). These two issues were partly solved upon the development of the higher-density SNP chip (Illumina BovineHD BeadChip; Rincon et al., 2011). This recently developed tool has shown its usefulness in detecting signatures of selection in dairy and beef cattle breeds (Utsunomiya et al., 2013; Kemper et al., 2014; Perez O'Brien et al., 2014; Xu et al., 2015; Chen et al., 2016) and in tropical-adapted cattle (Porto-Neto et al., 2014; Xu et al., 2015). More specifically, Utsunomiya et al. (2013) have reported for the first time in cattle the use of a composite mapping index “Meta-analysis of Selection Signals (*meta-SS*),” using the Z-transformation method “Stouffer's method” (Stouffer et al., 1949), to define footprints of positive selection related to meat and milk production traits.

Full genome sequencing has also been used in livestock species to detect signatures of positive selection, e.g., in chicken (Rubin et al., 2010), pig (Rubin et al., 2012; Frantz et al., 2015), sheep (Liu et al., 2016), and cattle (Liao et al., 2013; Qanbari et al., 2014; Choi et al., 2015). In Gir cattle, assessing the pooled heterozygosity of sliding windows has defined footprints of selection on genes associated with heat tolerance, and innate and adaptive immunity (Liao et al., 2013). In Fleckvieh cattle, genes associated with coat color and sensory perception (e.g., olfaction and taste) have also shown signatures of positive selection (Qanbari et al., 2014). The *PPP1R12A* gene involved in intramuscular fat content was also considered as a candidate of positive selection upon analyzing the full genome re-sequence of Hanwoo cattle (Choi et al., 2015).

Recently, we explored the genome of an indigenous EASZ from western Kenya population using genome-wide SNP data from the Illumina BovineSNP50 BeadChip v.1, and identified 24 candidate genome regions harboring signatures of positive selection (Bahbahani et al., 2015). However, given the restricted genome coverage of this tool (Matukumalli et al., 2009), these likely represent only a subset of the positively selected regions. We now analyze the autosomes of the same population for signatures of positive selection using two highly informative genome-wide dataset; genome-wide SNP genotypes obtained from the Illumina BovineHD BeadChip and full genome re-sequencing data. Furthermore, we include zebu × taurine populations from Uganda and Nigeria in our SNP genotyping analysis to assess the presence of commonly selected regions across these populations. Our aim is to provide a complete picture of the genome landscape of positive selection footprints in EASZ and in particular attend to untangle, at genome level, selection for the environmental challenges and the two components of animals fitness; reproduction and survivability. The identified regions were further explored to define possible causative variants responsible for the associated signatures of selection.

## Materials and methods

### Cattle populations, SNP genotyping, and quality control

Details of the cattle populations studied are presented at Table 1. They include 92 non-European introgressed small EASZ from the Western and Nyanza provinces of Kenya (KEASZ) as well as cattle from Uganda, Nigeria, Guinea, Europe, and India. They were genotyped for 777,962 SNPs mapped to the UMD3.1 bovine reference genome using the Illumina BovineHD Genotyping BeadChip.

**Table 1:** The studied cattle populations.

**Population abbreviation**	**Population name**	Population type^*^	**Population origin**	**Number of samples**
KEASZ	East African Shorthorn zebu	Small EASZ	Kenya	92
AG	Adamawa gudali	African zebu	Nigeria	25
AZ	Azawak	African zebu	Nigeria	2
BJ	Bunaji	African zebu	Nigeria	22
OR	Red bororo	African zebu	Nigeria	22
SO	Sokoto gudali	African zebu	Nigeria	19
WD	Wadara	African zebu	Nigeria	3
YK	Yakanaji	African zebu	Nigeria	12
MT	Muturu	African taurine	Nigeria	8
KR	Karamojong zebu	Large EASZ	Uganda	16
ZS	Serere zebu	Small EASZ	Uganda	13
AO	Ankole	Sanga	Uganda	25
NG	Nganda	Zenga	Uganda	23
NDM	N'Dama	African taurine	Guinea	24
HOL	Holstein-Friesian	European taurine	Europe	63
JER	Jersey	European taurine	Europe	36
NEL	Nelore	Asian zebu	India	35
GIR	Gir	Asian zebu	India	30

* *Types of African cattle populations following DAGRIS (2007)*.

Quality control (QC) analyses for 735,297 autosomal SNPs were conducted through the *check. marker* function implemented in the GenABEL package (Aulchenko et al., 2007) for R software version 2.15.1 (R development Core Team, 2012). SNPs with a minor allele frequency (MAF) <0.05 (*n* = 68,731) or call rate <95% (*n* = 18,667) were filtered out from the entire dataset. These include 1,712 SNPs that failed both criteria. In total, 649,611 SNPs were therefore retained. The ancestral allelic state for 373,005 SNPs [mean gap size = 6.7 kb and standard deviation (SD) = 12.1 kb] has been reported previously by Utsunomiya et al. (2013) following genotyping of three non-cattle *Bovinae* species: two *B. gaurus* (gaur), six *Bubalus bubalis* (water buffalo), and two *B. grunniens* (yak), with the fixed allele in the three species considered as ancestral. Only these SNPs were included in the downstream analyses.

Additional QC criteria included a minimum sample call rate of 95% and a maximum pairwise identity-by-state (IBS) of 95%, with the lower call rate animal eliminated from the high IBS pair. One ZS sample was excluded for having a low call rate, whilst 15 samples (two GIR, one NEL, four HOL, four JER, two AG, one AZ, and one WD) were excluded following the IBS criterion.

### Principle component analysis (PCA)

PCA were conducted using the *prcomp* function implemented in GenABEL package for R software. These analyses were carried out in three levels; (i) all cattle populations, (ii) all cattle populations excluding the European taurine cattle, and (iii) only the KEASZ, Uganda (UGN) and Nigeria (NGR) cattle.

### Estimation of Asian zebu autosomal ancestry proportion in African zebu cattle (admixture analysis)

Admixture analysis using ADMIXTURE 1.23 software (Alexander et al., 2009) with cross-validation and 200 bootstraps for *K* = 3 was conducted on the whole dataset to determine the European taurine, Asian zebu, and African taurine ancestries at genome-wide level and for each autosome separately. The output files were graphically displayed by the ggplot2 package (Wickham, 2009) for R software.

### Extended haplotype homozygosity (EHH)—based statistics (*Rsb* and *iHS*)

*Rsb* analyses (Tang et al., 2007) were conducted between each of the KEASZ, combined UGN cattle populations (AO, KR, NG, ZS) and combined NGR cattle populations (AG, AZ, BJ, OR, SO, WD, YK; Tijjani, 2013; Mbole-Kariuki et al., 2014) with the combined reference cattle populations (NEL, GIR, NDM, MT, HOL, JER) using the *rehh* package (Gautier and Vitalis, 2012) for R software. The standardized *Rsb*-values were normally distributed (Supplementary Figure 1), so a *Z*-test was applied to identify statistically significant SNPs under selection on KEASZ, UGN, and NGR cattle populations. One-sided upper-tail *P*-values were derived as *1-*Φ*(Rsb)* from the Gaussian cumulative density function Φ. Candidate regions were defined as having five adjacent SNPs, not separated by more than 500 kb, passing the threshold of −log_10_ *P* = 4.

*iHS* analyses (Voight et al., 2006) were conducted on KEASZ, combined UGN cattle and combined NGR cattle populations using the *rehh* package for the R software. This statistic was calculated for SNPs that passed the QC criteria and exhibited a within-population MAF of at least 0.05, since the algorithm of *iHS* has a limited power to calculate the statistic for fixed SNPs. As with *Rsb*, the standardized *iHS*-values followed a normal distribution (Supplementary Figure 1), so a two-tailed *Z*-test was applied to identify statistically significant SNPs under selection with either an unusual extended haplotype of ancestral or derived alleles relative to the genome. Two-sided *P*-values were derived as *1-2|*Φ*(iHS)-0.5|* from the Gaussian cumulative density function Φ. Candidate regions were defined as in *Rsb*.

As a prerequisite to the *Rsb* and *iHS* analyses, *fastPHASE* 1.4 (Scheet and Stephens, 2006) was used to phase the genotyped SNPs into the corresponding haplotypes using K10 and T10 criteria. Population label information was used to estimate the phased haplotype background.

### Inter-population change in SNP allele frequency (Δ*AF*)

Δ*AF* analysis investigates absolute allele frequency difference between two populations (Carneiro et al., 2014). In this analysis, the mean frequency of the first allele was estimated for KEASZ, combined UGN cattle and combined NGR cattle populations, separately (AF_pop1_). Likewise, the mean frequency of the first allele for each SNP was calculated for the combined reference cattle populations (AF_pop2_). The standardized values of the Δ*AF* (AF_pop1_–AF_pop2_) were normally distributed (Supplementary Figure 1), therefore a *Z*-test was applied to identify statistically significant SNPs showing higher allele frequency in the first population. Two-sided *P*-values were derived as *1-2|*Φ (standardized ΔAF)-0.5| from the Gaussian cumulative density function Φ. Candidate regions were defined as in *iHS* and *Rsb*.

### Meta-analysis of selection signals (*Meta-SS*)

The Stouffer method was used to combine the *P*-values obtained from *Rsb, iHS*, and Δ*AF*, for each analyzed set of populations, i.e., KEASZ, UGN, and NGR, in *meta-SS* analyses (Whitlock, 2005; Utsunomiya et al., 2013). Each value, for every SNP in each test, was transformed to a Z-score, Φ^−1^ (1-*P*-value), where Φ^−1^ is the inverse cumulative distribution function for a standard normal distribution. Then, the SNP-specific Z-scores were combined together according to the following equation: Z_i_ = (Z_*Rsb*_ + Z_*iHS*_ + Z_ΔAF_)/$\sqrt{k}$, where i and *k* are numbers of SNPs and tests, respectively. The resulting Z-scores were referred back to the standard normal distribution to obtain combined *P*-values [*P*-value = 1- Φ(Z_i_)], where is the cumulative distribution function for a standard normal distribution. Candidate regions were defined as having five adjacent SNPs not separated by more than 500 kb passing the threshold of −log_10_ *P* = 4.

### KEASZ whole genome sequencing analysis

A single pool of 10 unrelated KEASZ DNA samples were sequenced using an ABI SOLiD 4 genetic analyser (Supplementary Table 1). SOLiD 2 × 50 bp mate-paired libraries were constructed and sequenced in the Deep Seq facility at the University of Nottingham according to the manufacturer's instructions. Reads were mapped to the UMD3.1 bovine reference genome assembly (Elsik et al., 2009) using the LifeScope Genomic Analysis software 2.5.1 re-sequencing mapping pipeline (http://www.lifetechnologies.com/lifescope). SNPs and indels (insertions/deletions) were called using the diBayes package implemented in LifeScope. A minimum coverage of two uniquely mapped reads and two non-reference allele counts were required to call a variant. Additionally, a minimum read mapping quality of 20 (MAPQ20) and base quality of 20 were also implemented.

Pooled heterozygosity *Hp* of SNPs detected in the pooled 10 KEASZ full genome SOLiD sequences were calculated on 100 kb sliding windows with 10 kb incremental steps. Window sizes were extended by the number of uncovered bases to improve the accuracy of the calculation and consistency across windows. For each SNP in the window, the number of reads for the most and least frequent allele was counted (n_MAJ_ and n_MIN_, respectively). *Hp*-values were calculated using the following formula: *Hp* = 2 ∑ n_MAJ_ ∑ n_MIN_/(∑ n_MAJ_ + ∑ n_MIN_)^2^ (Rubin et al., 2010). The autosomal *Hp*-values were Z-transformed (ZHp = *Hp*–mean *Hp*/SD *Hp*) and a ZHp ≤ −4 was applied as a threshold to specify windows carrying selective sweep as in Liao et al. (2013). Overlapping candidate windows were merged into a single region.

### KEASZ exome enrichment and sequencing

The Agilent SureSelect^XT^ target enrichment kit (cat no. G7530-90004) was used for bovine exome sequence enrichment. It covers a total of ~45 Mb of the bovine sequence that composed of the coding regions in the UMD3.1 reference genome (coding regions from Refseq and Ensembl, no UTR “untranslated regions”) and microRNA. The exomes of a further 10 KEASZ samples (Supplementary Table 1) were sequenced in the Deep Seq facility at the University of Nottingham using an ABI SOLiD 5500 genetic analyser. Using the LifeScope Enrichment Sequencing Pipeline, the generated 75 bp reads were mapped to the UMD3.1 bovine reference genome. Supplementary Table 2 summarizes the number of aligned reads, average depth of coverage, and percentage of reference exome for each sample. The same variants calling criteria used in the KEASZ whole genome sequence were implemented for the exome analysis except that reads with MAPQ ≥ 30 and base quality of 30 were used.

### African taurine (N'Dama and Muturu) genome sequence analysis

To infer about the origin of the variants identified in the KEASZ selected regions, we analyzed 10 N'Dama cattle full genome sequences from Guinea available at the GenBank with the Bioproject accession number PRJNA312138 (Kim et al., 2017), and 10 Muturu cattle genome sequences from South East Nigeria. For the later, genomic DNA was extracted using Macherey-Nagel NucleoSpin® Tissue DNA extraction kit, according to manufacturer's protocol, from 10 ear tissue samples. One hundred and fifty bp paired-end libraries were constructed and sequenced using Illumina Hiseq2500 platform (Illumina, USA). Sequence reads were mapped to the UMD3.1 bovine reference genome assembly using the bwa-mem option of Burrows-Wheeler Alignment tool (BWA) version 0.7.5a (Li and Durbin, 2010; Supplementary Table 3). Genome Analysis Toolkits (GATK) version 3.4.0 (McKenna et al., 2010) was used for base quality recalibration, indel realignment, PCR duplicate removal and variants (SNPs and indels) calling, using Haplotypecaller function, according to GATK best practices recommendations (DePristo et al., 2011; Van der Auwera et al., 2013). A minimum read mapping quality of 50 (MAPQ50) and base quality of 30 were set as two criteria in variant calling.

### Candidate selected regions characterization

Protein-coding and RNA genes mapped within the candidate regions, based on the UMD3.1 bovine reference genome annotation, were processed using the functional annotation tool implemented in *DAVID* Bioinformatics resources 6.7 to determine the over-represented (enriched) functional terms (Huang da et al., 2009a,b). An enrichment score of 1.3, which is equivalent to the Fisher exact test *P* = 0.05, was used as a threshold to define the significantly enriched functional terms in comparison to the whole bovine reference genome background. The list of genes mapped on the UMD3.1 reference bovine genome was obtained from the *Ensembl Genes 86* database (Flicek et al., 2013) using the *BioMart* tool (Kinsella et al., 2011). The bovine Quantitative Trait Loci (QTL) and their UMD3.1 genome coordinates were downloaded from the cattle QTL database (http://www.animalgenome.org/cgi-bin/QTLdb/BT/index). The *intersectBed* function from the *BedTools* software was used to overlap these QTL with the identified KEASZ candidate regions (Quinlan and Hall, 2010). Candidate regions lacking annotated genes (protein-coding, RNA, and pseudogenes) based on UMD3.1 bovine reference genome were also identified. The *intersectBed* function from the *BedTools* software was also used to overlap these candidate regions with transcription factors binding sites identified previously on the bovine reference genome by Bickhart and Liu (2013).

Variants (SNPs and indels) in the genes within the genome-wide SNP and *Hp* overlapping candidate regions were annotated using the variant effect predictor tool (McLaren et al., 2016) based on *Ensembl variants 86* databases. Comparisons with the previously discovered bovine variants listed in the dbSNP database (Sherry et al., 2001) classified these variants into KEASZ-specific (novel) and general bovine variants. The identified missense, splice region SNPs, frameshift and splice region indels were cross-checked with variants identified in the KEASZ exome data and with N'Dama and Muturu full genome sequences.

Candidate genes within the overlapping genome-wide SNP and *Hp* candidate regions were selected based on their biological function, e.g., immunity, reproduction, fertility, heat tolerance and anatomical development. Variants (SNP and indels) within these genes were defined and the biological effect of the missense SNPs were predicted by the online tool PolyPhen-2 (Adzhubei et al., 2010).

### Estimation of excess-deficiency in Asian zebu ancestry at candidate regions

LAMP software version 2.4 (Sankararaman et al., 2008) was used to estimate the Asian zebu and African taurine ancestry proportions of the high-density genotyped SNPs in the KEASZ samples. The genome-wide autosomal zebu ancestry proportion of 70% was obtained from the admixture proportions α of the ADMIXTURE analyses. An estimated number of 500 generations was set for the beginning of the zebu-taurine admixture in light of our current knowledge of zebu arrival on the continent, assuming a generation time of 6 years (Keightley and Eyre-Walker, 2000). A uniform recombination rate of 1 cM = 1 Mb was assumed. The average excess/deficiency in Asian zebu ancestry at each SNP (ΔAZ) was calculated by subtracting the average estimated Asian zebu ancestry of the SNP from the average estimated Asian zebu ancestry of all SNPs. The median ΔAZ for the significant SNPs of KEASZ *meta-SS* analysis within candidate regions was considered.

## Results

### The admixed genome structure of African cattle populations

**Figure 1:**
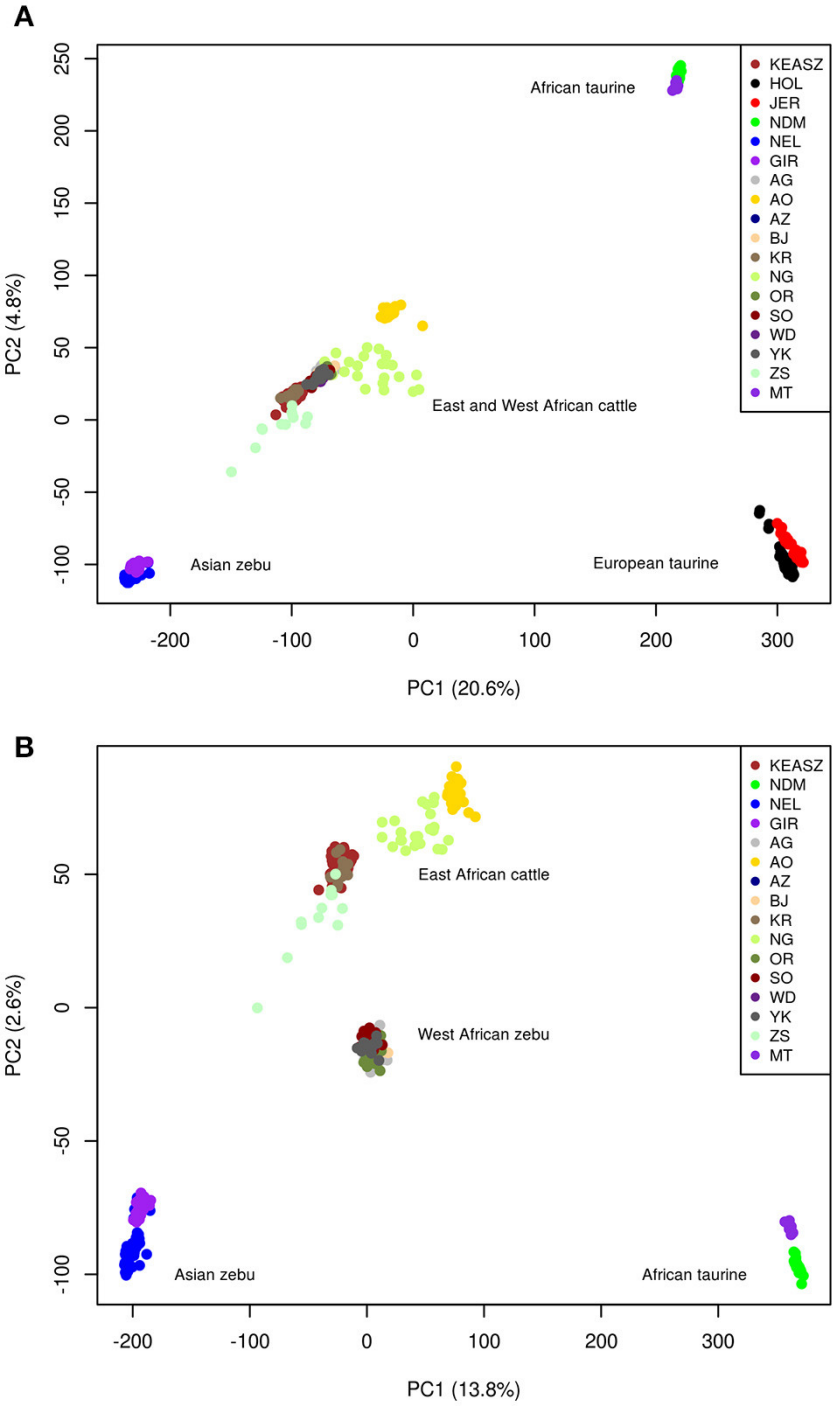
Plots of the highest two principle components resulted by analyzing autosomal SNPs in **(A)** all cattle populations included in this study, and **(B)** all cattle excluding European taurine populations.

The PCA plot on all cattle populations using the filtered autosomal SNPs dataset (Figure 1A) reveals the already described triangle-like 2-dimentional global organization of cattle genetic diversity (Gautier et al., 2010). The first component, which accounts for 20.6% of the total variation, separates the European (HOL and JER) and African (NDM and MT) taurine populations from the Asian zebu cattle (NEL and GIR). The second component, which accounts for 4.9% of the total variation, separates the African taurine cattle from the other cattle populations. All the East (KEASZ and UGN) and West (NGR) African cattle populations analyzed are positioned between the African taurine and Asian zebu cattle supporting zebu × taurine genome admixture.

The PCA conducted on Asian zebu (NEL and GIR), African taurine (NDM and MT), East African (KEASZ and UGN) and West African (NGR) cattle populations (Figure 1B) shows genetic differentiation within the East African cattle (KEASZ and UGN) cattle and between the East and West African (NGR) cattle. The first component, which explains 13.9% of the total variation, separates Asian zebu from African taurine. Whilst, the second component, which explains 2.6% of the total variation, separate West African zebu cattle (NGR) and the East African (KEASZ and UGN) cattle. A PCA on KEASZ, UGN, and NGR cattle only, separates first the East (KEASZ and UGN) and West (NGR) cattle populations (*PC1* = 2.99%), while PC2 (2%) reveal a much higher genetic heterogeneity within and across East African cattle populations compared to the West African ones (Supplementary Figure 2).

**Figure 2:**
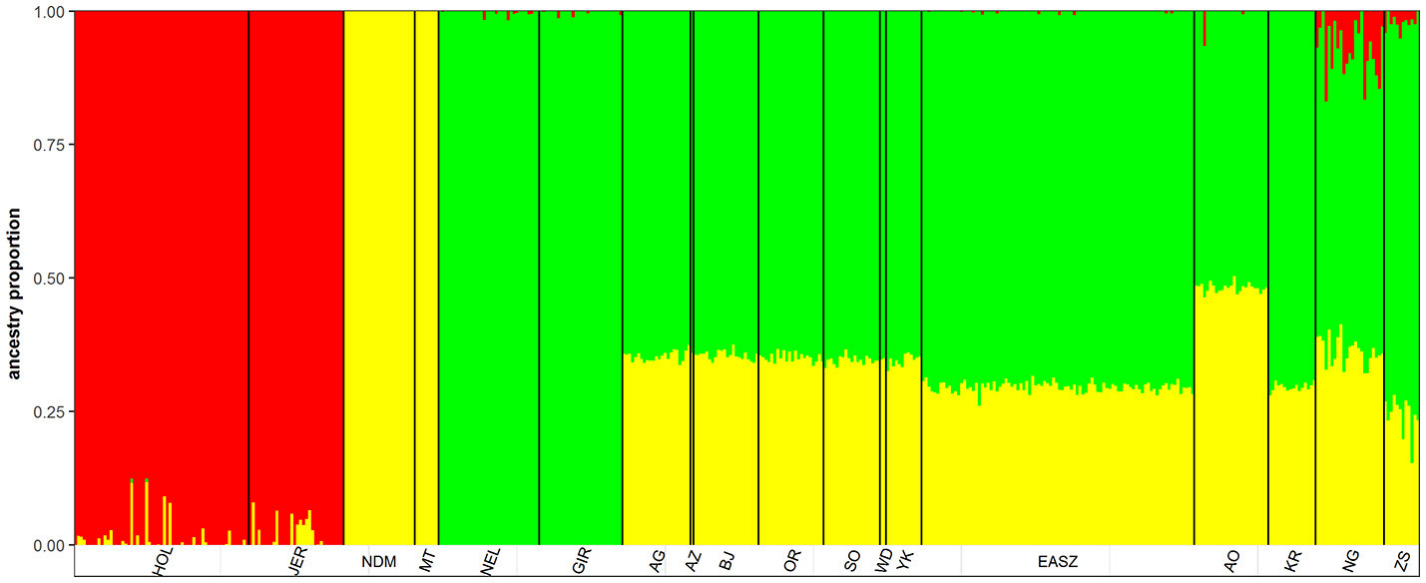
ADMIXTURE bar plot of the whole dataset at *K* = 3.

The admixture analysis indicates a relatively even admixed genome of Asian zebu and African taurine ancestries across KEASZ animals with an estimated genetic proportion of 0.7 ± 0.01 SD and 0.30 ± 0.01 SD, respectively. The same even admixed proportion is present across the Karamajong zebu cattle from Uganda, while the Serere zebu has an estimated 0.74 ± 0.04 SD and 0.24 ± 0.04 SD zebu × taurine genetic proportions, with minor European taurine ancestry (0.02 ± 0.01 SD). The genome of the Ankole cattle has a larger African taurine genetic proportion (0.48 ± 0.01 SD). Nganda cattle has an admixed genome of 0.57 ± 0.04 SD Asian zebu and 0.36 ± 0.03 SD African taurine ancestries, it also carries low level of European taurine ancestry (0.07 ± 0.05 SD) (Figure 2). This pattern of admixture is also observed in the cattle of Nigeria but with higher average African taurine ancestry (0.35 ± 0.01 SD) and lower zebu ancestry (0.65 ± 0.01 SD) than the East African cattle, except Ankole. No European introgression was detected in the West African zebu cattle analyzed.

Variation in Asian zebu ancestry is observed among the autosomes of the East (KEASZ and UGN) and West African (NGR) cattle populations (Supplementary Figure 3, Supplementary Table 4) with increase or decrease (mean ± one SD) in Asian zebu ancestry proportions (Table 2). A strong correlation in the zebu ancestry autosomal variation is observed when comparing KEASZ with East (UGN) and West (NGR) cattle populations (*P* < 0.00001; Supplementary Figure 4).

**Table 2:** Autosomes with substantial increase or decrease in Asian zebu ancestry among the East and West African cattle populations.

**BTA**	**East African cattle**					**West African zebu cattle**
	**KEASZ**	**AO**	**KR**	**ZS**	**NG**	**NGR**
	**Mean = 0.69, SD = 0.05**	**Mean = 0.5, SD = 0.06**	**Mean = 0.69, SD = 0.05**	**Mean = 0.72, SD = 0.05**	**Mean = 0.55, SD = 0.06**	**Mean = 0.64, SD = 0.04**
2						
3	+		+	+		
4					+	
5			+	+		+
6						
7	+	+	+	+	+	+
8						
9	−	−		−	−	
11		−				+
12		+		+		
13	+	+	+	+	+	+
14						
15				−	−	
16	+	+	+	+	+	
17	−	−	−	−	−	−
18						+
19	+	+	+	+	+	+
20	−	−				
21		−		−		−
22		−				
23	−	−	−	−		−
25	−		−		−	
26					+	+
27	−	−			−	−
28	−	−	−		−	−
29	−	−	−	−	−	−

*KEASZ, East African shorthorn zebu from western Kenya; AO, Ankole; KR, Karamojong zebu; NG, Nganda; ZS, Serere zebu; NGR, West African zebu cattle from Nigeria. +: Substantial increase in Asian zebu ancestry (≥mean + one SD). –: Substantial decrease in Asian zebu ancestry (≤mean – one SD)*.

### Signatures of positive selection

#### High density genome-wide SNP analysis

**Figure 3:**
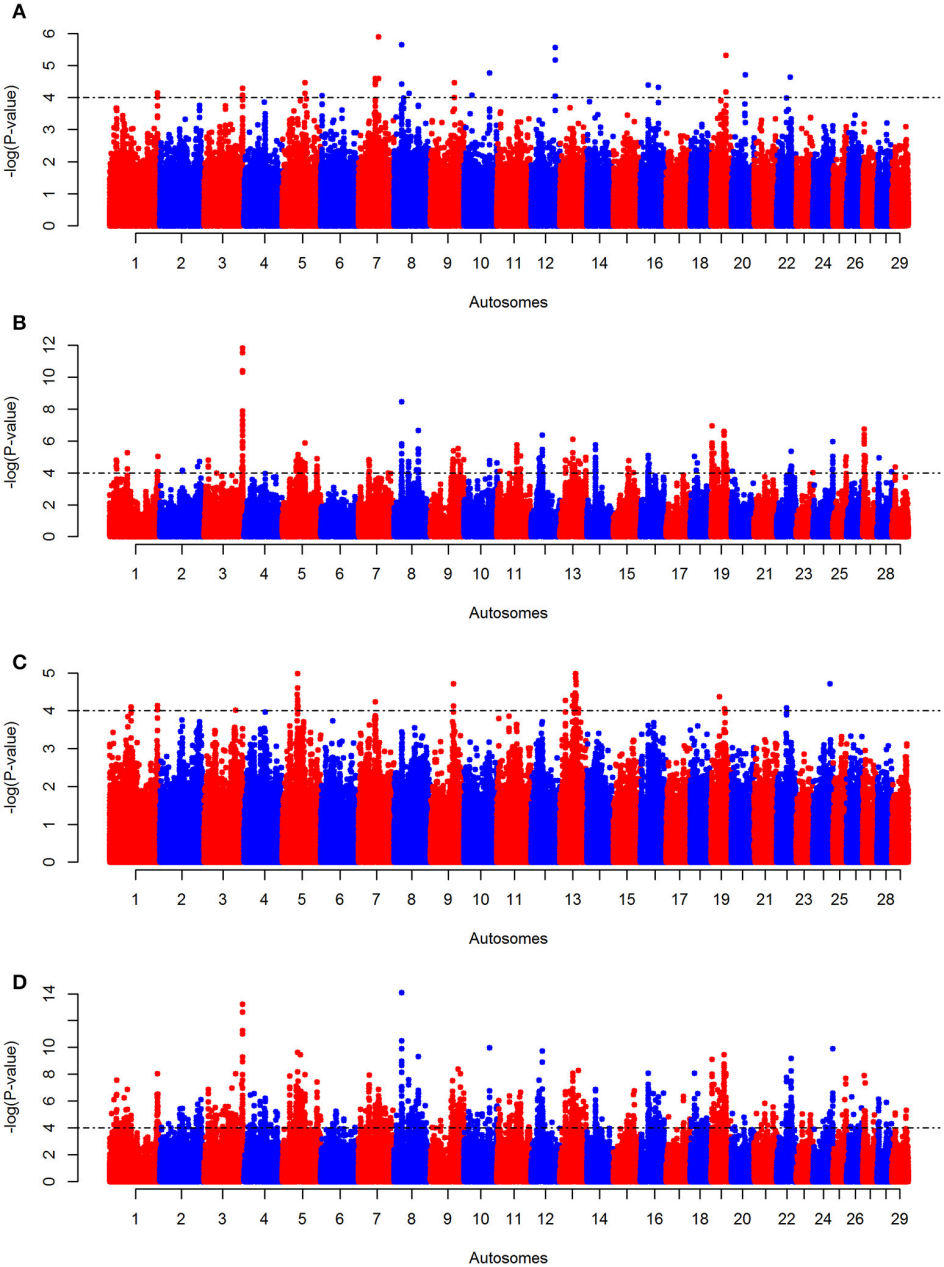
Manhattan plots for autosomal **(A)** KEASZ *iHS*, **(B)** *Rsb*, **(C)** Δ*AF*, and **(D)** *meta-SS* analyses between KEASZ and combined reference populations (Holstein-Friesian, Jersey, N'Dama, Muturu, Nelore and Gir). Threshold is set at–log_10_ *P* = 4.

The *iHS, Rsb*, and Δ*AF* analyses conducted on KEASZ reveal one, 19 and six autosomal candidate regions harboring signatures of positive selection, respectively (Figures 3A–C, Supplementary Table 5). As the tests follow normal distributions (Supplementary Figure 1) and their genome-wide average *P*-values are weakly correlated (Pearson correlation coefficient *r* ≤ 0.228; Supplementary Table 6), the *P*-values of each SNP for the three tests were combined in a *meta-SS* analysis revealing 98 candidate regions (Figure 3D, Supplementary Table 5). All the candidate regions defined by each individual test are also included as candidate regions by the *meta-SS* analysis, with the exception of three regions on BTA 13 identified by Δ*AF* analysis only. Seventy-seven new candidate regions are detected after combining the *P*-values of the three tests.

In East African cattle populations from Uganda (UGN), the three tests (*iHS, Rsb* and Δ*AF*) reveal six, 25 and three autosomal candidate regions, respectively (Supplementary Figures 5A–C, Supplementary Table 7). The same analyses on the zebu cattle populations from Nigeria (NGR) identify four, 22 and four autosomal candidate regions, respectively (Supplementary Figures 6A–C, Supplementary Table 8). After combining the tests *P*-values, 86 and 97 regions are considered as candidate regions for positive selection in UGN and NGR cattle populations, respectively (Supplementary Figures 5D, 6D, Supplementary Tables 7, 8).

#### Comparison with African cattle from Uganda and Nigeria

A total of 32 KEASZ candidate regions are shared with the UGN cattle populations. We classify them as East African candidate regions. Twenty-two regions are also shared across the three comparisons and they are classified as East and West African candidate regions (Table 3). The cross-validation of candidate regions between the three sets of zebu × taurine admixed cattle populations (KEASZ, UGN, and NGR) allow us to fine map the size of 18 candidate regions. As indicated in Table 4, this approach allows us to narrow the size of these candidate regions to 94–894 kb, with the largest reduction in size (~1.9 Mb) observed for a candidate region on BTA 12 (Table 4).

**Table 3:** Shared candidate regions obtained by the genome-wide SNP analyses.

**BTA**	**Position (UMD 3.1)**		**Value^c^**	**ΔAZ^a^**	**Other studies^b^**
	**Start**	**Stop**			
**(A) EAST AFRICAN CANDIDATE REGIONS**					
2	125,159,084	125,994,861	5.17	0.07390136	Gautier and Naves, 2011
3	34,254,043	34,727,876	5.52	0.09020536	
3	120,601,191	121,238,836	13.22	0.07390136	
5	23,652,016	24,338,695	7.86	0.09020536	Gautier et al., 2009
5	109,303,999	109,688,098	7.43	0.07933636	
7	61,232,987	61,396,966	5.90	−0.1217506	Gautier et al., 2009
8	23,344,221	23,663,852	14.08	−0.0402296	
8	65,373,897	65,634,601	5.55	0.05487936	
9	69,198,185	69,406,467	7.53	0.07933636	
9	73,280,867	74,185,868	5.73	0.10107536	
**9**	**76,289,561**	**76,853,587**	5.06	**0.13911836**	
9	94,121,197	94,242,831	8.06	0.11194436	Larkin et al., 2012^*^
10	80,515,703	80,796,559	9.99	0.07118386	
11	38,402,190	39,743,107	6.37	−0.0076206	Gautier et al., 2009; Kemper et al., 2014^*^
11	71,387,248	72,221,099	6.33	−0.1299031	
12	21,086,969	21,254,061	6.17	−0.0565336	
12	24,843,013	25,658,768	7.56	0.11194486	
12	35,689,908	36,746,504	9.74	0.05759636	
13	39,579,929	41,356,847	8.07	0.08748786	Perez O'Brien et al., 2014
**13**	**47,532,424**	**48,142,997**	**4.22**	**0.14455335**	
13	49,433,476	49,762,965	4.99	0.12281436	Porto-Neto et al., 2013
**13**	**50,616,630**	**50,837,529**	5.68	**0.18259636**	
13	58,273,562	58,599,491	8.29	0.06846636	Flori et al., 2014; Kemper et al., 2014^*^
14	28,186,226	28,430,215	6.86	0.08477036	
**16**	**26,979,772**	**27,160,301**	5.77	**0.18259636**	
**16**	**46,869,577**	**47,614,377**	6.08	**0.18803136**	Liao et al., 2013; Kemper et al., 2014^*^
**19**	**3,337,282**	**3,823,638**	5.19	−**0.1815336**	
19	9,515,063	9,780,078	7.24	−0.0945776	
19	39,330,233	39,519,992	4.68	0.10107536	
19	40,045,779	40,808,559	9.48	0.03585736	Gautier et al., 2009
**21**	**60,026,698**	**60,449,172**	5.55	**0.17716236**	
**22**	**29,533,544**	**30,366,810**	7.76	**0.15542236**	
**(B) EAST AND WEST AFRICAN CANDIDATE REGIONS**					
**1**	**149,547,998**	**149,960,460**	6.50	**0.1771624**	
2	70,314,631	71,161,113	5.45	0.1119444	Gautier et al., 2009; Liao et al., 2013
**3**	**76,084,701**	**76,413,468**	6.6	−**0.1652296**	Kemper et al., 2014^*^
3	98,862,402	99,283,161	8.05	0.1200969	
5	43,834,751	44,574,214	7.12	0.1065094	
5	48,477,903	49,212,943	9.64	0.1282494	Gautier et al., 2009; Ramey et al., 2013^*^; Perez O'Brien et al., 2014; Xu et al., 2015^*^
5	62,272,683	62,587,423	5.69	−0.0837076	Kemper et al., 2014^*^
7	32,640,500	33,093,884	7.17	0.0956404	
7	50,281,923	50,670,070	4.49	0.1336834	
7	62,551,178	62,782,874	6.89	0.0793364	
**11**	**62,343,547**	**62,548,419**	5.76	**0.1662924**	
**12**	**28,949,354**	**29,151,436**	4.23	−**0.2141426**	Gautier et al., 2009; Gautier and Naves, 2011; Liao et al., 2013; Porto-Neto et al., 2013^*^; Flori et al., 2014
**13**	**18,132,557**	**18,320,265**	6.7	**0.1662924**	
16	25,389,029	25,540,339	8.09	0.1173794	
16	50,610,769	50,762,363	4.69	0.0412924	Gautier and Naves, 2011
19	2,568,979	2,765,065	9.1	−0.0619686	
19	27,004,483	27,143,239	6.66	0.1228144	
19	44,788,419	44,924,467	6.3	0.1336834	
**19**	**46,580,102**	**46,673,984**	8.05	**0.1391184**	
21	33,590,777	33,696,403	5.03	−0.0021856	
22	45,231,901	46,126,149	9.20	0.1119444	Gautier et al., 2009; Flori et al., 2014
24	61,972,128	62,530,799	9.90	0.0032489	

***(A)** East African candidate regions [KEASZ and Uganda (UGN)] **(B)** East and West African candidate regions [KEASZ, Uganda (UGN) and Nigeria (NGR)]*.

a *ΔAZ = estimated excess/deficiency of the Asian zebu ancestry proportion*.

b *The candidate regions were cross-referenced with the ones obtained previously on tropical-adapted cattle and commercial breeds*.

c *− log(P-value) of highest KEASZ SNP within the region*.

*Bold (deviation by more than ±1 standard deviation from the mean ΔAZ)*.

* *Studies on commercial breeds*.

**Table 4:** Fine mapping of KEASZ candidate regions.

**KEASZ candidate regions size (UMD 3.1)**				**Overlapping East and West African candidate regions (UMD3.1)**				**Reduction in size (bp)**
**BTA**	**Start**	**End**	**Size**	**BTA**	**Start**	**End**	**Size**	
1	149,241,884	149,992,523	750,639	1	149,547,998	149,960,460	412,462	338,177
2	70,314,631	71,161,113	846,482	2	70,314,631	71,161,113	846,482	None
3	76,084,701	76,781,970	697,269	3	76,084,701	76,413,468	328,767	368,502
3	98,862,402	99,422,213	559,811	3	98,862,402	99,283,161	420,759	139,052
5	43,230,619	44,574,214	1,343,595	5	43,834,751	44,574,214	739,463	604,132
5	48,477,903	49,268,610	790,707	5	48,477,903	49,212,943	735,040	55,667
5	62,272,683	62,659,987	387,304	5	62,272,683	62,587,423	314,740	72,564
7	31,748,136	33,875,610	2,127,474	7	32,640,500	33,093,884	453,384	1,674,090
7	50,281,923	50,809,190	527,267	7	50,281,923	50,670,070	388,147	139,120
7	62,415,406	63,117,931	702,525	7	62,551,178	62,782,874	231,696	470,829
11	61,877,437	62,548,419	670,982	11	62,343,547	62,548,419	204,872	466,110
12	27,050,192	29,151,436	2,101,244	12	28,949,354	29,151,436	202,082	1,899,162
13	18,130,223	18,421,481	291,258	13	18,132,557	18,320,265	187,708	103,550
16	24,517,859	25,540,339	1,022,480	16	25,389,029	25,540,339	151,310	871,170
16	50,610,769	50,762,363	151,594	16	50,610,769	50,762,363	151,594	None
19	2,568,979	2,765,065	196,086	19	2,568,979	2,765,065	196,086	None
19	26,909,816	27,143,239	233,423	19	27,004,483	27,143,239	138,756	94,667
19	44,788,419	45,414,418	625,999	19	44,788,419	44,924,467	136,048	489,951
19	46,031,543	46,786,391	754,848	19	46,580,102	46,673,984	93,882	660,966
21	33,590,777	33,696,403	105,626	21	33,590,777	33,696,403	105,626	None
22	45,102,551	46,400,273	1,297,722	22	45,231,901	46,126,149	894,248	403,474
24	61,008,938	62,530,799	1,521,861	24	61,972,128	62,530,799	558,671	963,190

Ten of the East African candidate regions intersect with regions under positive selection identified previously (Gautier et al., 2009; Gautier and Naves, 2011; Larkin et al., 2012; Kemper et al., 2014; Xu et al., 2015). Nine of these regions were identified in tropical-adapted cattle populations, such as Creole, Borgou, and Guzerat. Whilst four were found to be under positive selection in commercial cattle breeds, e.g., Holstein, Shorthorn, and Charolais.

For the 22 shared East and West African candidate regions, five were found to be under selection in tropical-adapted cattle (e.g., Gir, Creole, and Borgou) and four in commercial breeds (e.g., Angus, Holstein, and Shorthorn; Table 3).

#### Functional characterization of high density genome-wide SNP candidate regions

The 101 KEASZ candidate regions included 1,024 genes based on the UMD3.1 bovine reference genome annotation (Supplementary Table 9). These genes cluster into 110 functional clusters following DAVID functional term clusters enrichment analysis. Six of these clusters are significantly enriched relative to the bovine genome as indicated in Table 5. Candidate regions shared between KEASZ and UGN cattle populations harbor 309 genes (Supplementary Table 9). They are grouped into 32 functional term clusters, in which three are significantly enriched (Table 5). For candidate regions shared across East (KEASZ and UGN) and West (NGR) African cattle populations, 87 genes are identified. They are grouped into 10 functional term clusters, in which a single cluster, associated with immune response to bacterial infection, is significantly enriched (Table 5).

**Table 5:** Significantly enriched functional term clusters in KEASZ, East African (KEASZ and UGN), and East and West African (KEASZ, UGN, and NGR) candidate regions.

**Functional term cluster**	Score^*^
**KEASZ**	
Intermediate protein filaments and keratin	4.16
Cytoskeleton	2.4
Enzyme inhibitor activity	2.23
Cell-substrate (e.g., extracellular matrix) junction	1.71
Cell-cell junction and sensory perception	1.5
Immunity signals	1.3
**EAST AFRICAN CANDIDATE REGIONS**	
Nucleoplasm and nuclear lumen	1.76
Cell-cell junction	1.61
Defence response to bacteria	1.48
**EAST AND WEST AFRICAN CANDIDATE REGIONS**	
Defence response to bacteria	1.83

* *Enrichment score following DAVID analysis (a score equals to 1.3, equivalent to Fisher exact test P-value = 0.05, was used as a significant threshold)*.

Seven KEASZ candidate regions are classified as gene desert regions. Two of these regions are also identified in UGN cattle populations and none with NGR cattle (Supplementary Table 10). No transcription factors binding sites identified on cattle genome by Bickhart and Liu (2013) are overlapping with any of these gene desert regions.

#### KEASZ full genome sequencing analysis

The 10 pooled KEASZ full genome sequences generated a total of 615,413,240 reads with MAPQ ≥ 20 (0.1% probability of incorrect alignment) mapped on the UMD3.1 bovine reference autosomes. These MAPQ20 reads covered ~97% of the reference autosomes with an average of 11 times depth of coverage. SNP calling using LifeScope diBayes package identified a total of 10,466,699 SNPs (8,114,664 heterozygotes and 2,352,035 homozygotes).

**Figure 4:**
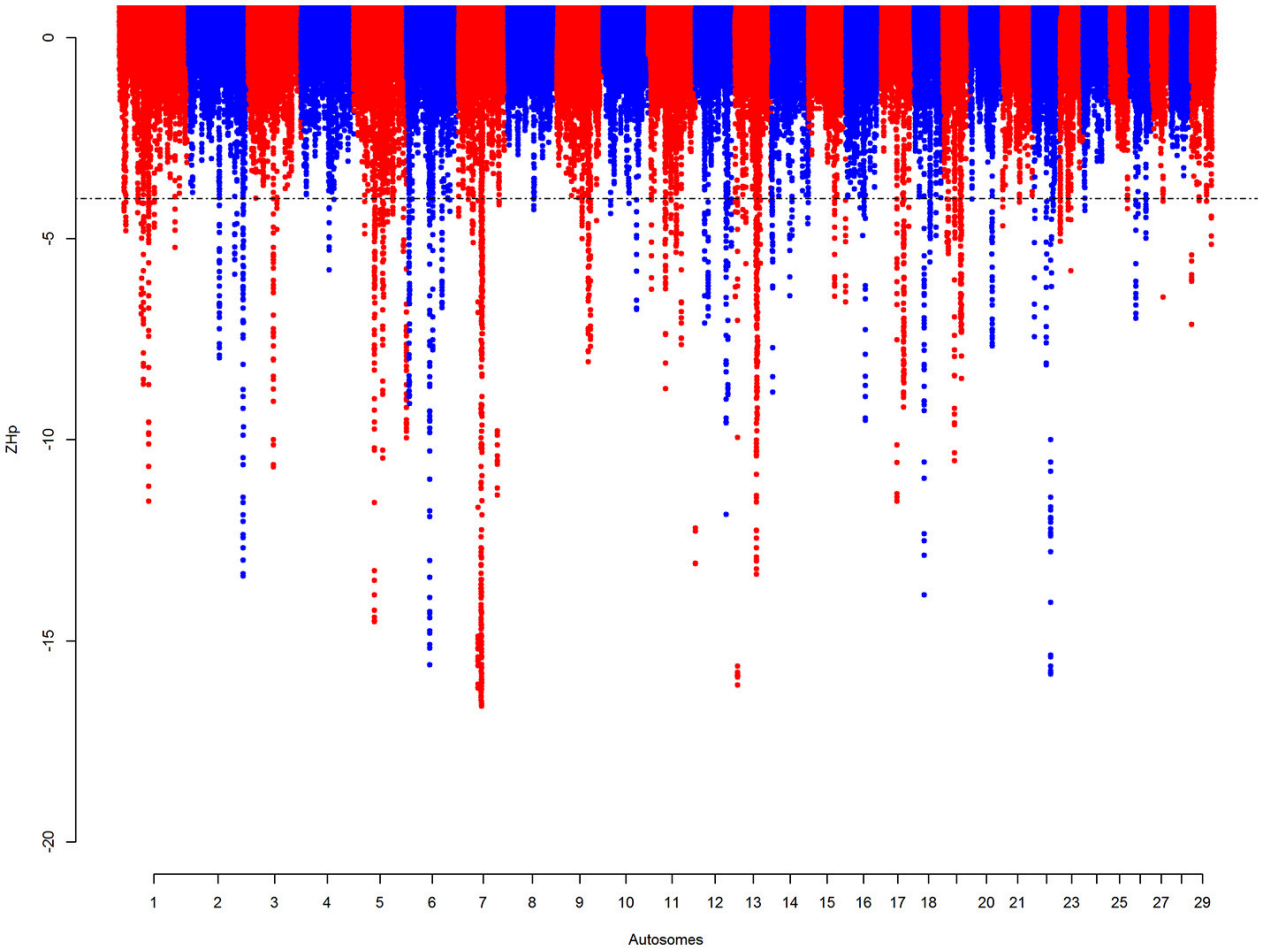
Manhattan plot for the autosomal *Hp* analyses on KEASZ. Each point represents a 100 kb window. The significant threshold is set at ZHp = −4.

Regions with signatures of selective sweep were defined by assessing the pooled SNPs heterozygosity *Hp* of 100 kb windows, as in Liao et al. (2013), incremented by 10 kb. Supplementary Figure 7 shows the distribution of SNPs in the 100 kb autosomal windows with a mean of 297 SNPs per window. The mean *Hp*-value is 0.42 (SD = 0.025). Out of the total 250,930 windows, 1,825 (~0.73%) have a ZHp score of ≤ −4 merged into 165 autosomal candidate sweep regions (Figure 4, Supplementary Table 11). The largest region, ~2 Mb in size, is on BTA 7 (51.4–53.4 Mb). This region contains windows with the lowest ZHp-value (ZHp = −16.6).

Based on the annotated UMD3.1 bovine reference genome, 518 genes are found within 133 of these sweep regions (Supplementary Table 12). DAVID analyses were conducted in two levels including: (i) genes within KEASZ *Hp* candidate regions and (ii) genes within all KEASZ candidate regions from the SNPs and *Hp* analyses combined together. The first DAVID analysis identifies 57 functional term clusters with six significantly enriched clusters (Table 6). Whilst, the second one defines 148 clusters with six significantly enriched clusters (Table 6).

**Table 6:** Significantly enriched functional term clusters of the genes mapped within **(A)** *Hp* candidate sweep regions. **(B)** Combined KEASZ candidate regions (SNPs and *Hp* analyses).

**Functional term cluster**	Score^*^
**A**	
Cell-cell adhesion	4.52
Response to hormones stimuli (e.g., growth hormones)	1.63
Regulation of T and B cells proliferation and activation	1.42
Regulation of cell cycle and organism growth	1.34
Chemotaxis	1.34
Immunity development	1.31
**B**	
Intermediate protein filaments and keratin	3.23
Enzyme inhibitor activity	2.34
Cell-cell adhesion	1.98
Protein transport and localization	1.98
Cytoskeleton	1.45
Nuclear lumen and nucleoplasm	1.34

* *Enrichment score following DAVID analysis (a score equals to 1.3, equivalent to Fisher exact test P-value = 0.05, was used as a significant threshold)*.

A total of 32 candidate regions are gene deserts (Supplementary Table 10). Five of these regions were present in UGN cattle and one in NGR cattle (genome-wide HD SNPs analysis). None of transcription factors binding sites, identified by Bickhart and Liu (2013), are overlapping with the gene desert regions identified here.

#### Overlapping candidate sweep regions between genome-wide HD SNP and Hp full genome sequence analyses

Among the 165 candidate *Hp* sweep regions, 35 regions overlap with the genome-wide HD SNP candidate regions of KEASZ. These include 25 regions also revealed by the SNP *meta-SS* analysis in UGN cattle populations, in which seven regions were also shared between the East (KEASZ and UGN) and West African (NGR) cattle populations (Table 7). Also, our genome sequence analysis reveals 101 candidate regions not previously identified to be under positive selection in other studied cattle populations (Supplementary Table 11).

**Table 7:** The overlapping candidate sweep regions between KEASZ *Hp* and genome-wide HD SNP analyses.

**BTA**	**Start**	**Stop**	**Mean ZHp**	**ΔAZ^a^**	**Other studies^b^**
**1**	**54,880,001**	**55,141,728**	−6.56	**0.139118**	
1	55,150,001	55,253,859	−4.12	NA	
2^**^	70,570,001	70,811,366	−6.68	0.114662	Gautier et al., 2009; Liao et al., 2013; Kemper et al., 2014^¥^
2^**^	70,990,001	71,191,313	−5.27	0.111944	Gautier et al., 2009; Liao et al., 2013; Kemper et al., 2014^¥^
2^*^	**125,300,001**	**125,620,820**	−8.12	**0.144553**	Gautier et al., 2009
2^*^	125,640,001	126,083,262	−7.66	0.073901	Gautier et al., 2009
5^**^	48,610,001	49,021,113	−5.01	0.125531	Liao et al., 2013; Kemper et al., 2014^¥^; Xu et al., 2015; Perez O'Brien et al., 2014
5^**^	49,120,001	49,241,076	−4.47	0.128249	Liao et al., 2013; Kemper et al., 2014^¥^; Perez O'Brien et al., 2014
7^*^	31,740,001	31,897,059	−4.54	0.08477	Flori et al., 2014
7^*^	33,100,001	33,293,306	−4.43	−0.00219	
**7**	**51,360,001**	**53,362,761**	−10.79	**0.198901**	Gautier et al., 2009; Liao et al., 2013; Porto-Neto et al., 2013; Qanbari et al., 2014^¥^
9^*^	**73,890,001**	**74,081,863**	−5.45	**0.188031**	
9^*^	76,600,001	76,876,188	−6.12	0.09564	
11^*^	39,240,001	39,530,799	−5.93	0.106509	Gautier et al., 2009; Kemper et al., 2014^¥^
11^*^	39,550,001	39,683,044	−4.38	NA	Gautier et al., 2009; Kemper et al., 2014^¥^
11	75,230,001	75,441,012	−6.40	0.054879	
12	20,870,001	21,021,506	−5.64	0.079336	Gautier et al., 2009
12^*^	21,130,001	21,320,859	−4.63	−0.05653	Gautier et al., 2009
12^**^	**29,110,001**	**29,438,417**	−5.83	−**0.21414**	Gautier et al., 2009; Gautier and Naves, 2011; Liao et al., 2013; Porto-Neto et al., 2013; Flori et al., 2014
13^*^	**47,980,001**	**48,164,495**	−4.99	**0.204336**	
13^*^	**48,650,001**	**49,056,444**	−10.07	**0.20977**	Porto-Neto et al., 2013
13^*^	49,340,001	49,551,378	−5.80	−0.00762	Porto-Neto et al., 2013
13^*^	**49,590,001**	**49,844,283**	−6.78	**0.182596**	Porto-Neto et al., 2013
13^*^	**50,240,001**	**50,852,056**	−7.32	**0.182596**	
13	55,510,001	55,623,671	−4.48	0.128249	
13	82,010,001	82,111,606	−4.06	0.09564	
19^*^	9,500,001	9,631,079	−4.72	−0.09458	
19^**^	26,890,001	27,154,002	−7.60	0.122814	Gautier et al., 2009
19^*^	39,270,001	39,422,844	−4.42	0.014119	
19^*^	40,490,001	40,714,976	−4.60	0.035857	
**19**	**40,960,001**	**41,450,870**	−5.98	**0.144553**	
**19**	**42,890,001**	**43,122,753**	−6.09	**0.149988**	Chen et al., 2010
**19**	**43,140,001**	**43,341,262**	−6.53	**0.141836**	Chen et al., 2010
22^*^	**30,030,001**	**30,260,687**	−6.05	**0.188031**	
22^**^	**45,220,001**	**45,370,457**	−4.17	**0.149988**	Gautier et al., 2009; Chen et al., 2010; Flori et al., 2014

a *ΔAZ = estimated excess/deficiency of the Asian zebu proportion*.

b *The candidate regions were cross-referenced with the ones obtained previously on tropical-adapted cattle and commercial breeds*.

*Bold (deviation by more than ±1 standard deviation from the autosomal mean ΔAZ)*.

¥ *Commercial breeds studies*.

*NA: No SNPs passed–log_10_ (P-value) = 4 of EASZ meta-SS analysis*.

* *Specific to East African cattle populations (KEASZ and Uganda)*.

** *Shared between East (KEASZ and Uganda) and West (Nigeria) African populations*.

Within the 35 overlapping candidate regions, 185 genes are identified (Supplementary Table 13). DAVID analysis indicates 23 functional clusters with two significantly enriched functional clusters: response to hormone stimulus and signaling pathway (enrichment score = 2.07), and transcription regulation (enrichment score = 1.3). Also worth mentioning is a functional cluster associated with the immune system development and regulation, although it does not reach the 1.3 threshold (enrichment score = 1.24).

For the 25 East African overlapping sweep regions, 103 genes are found (Supplementary Table 13). They are grouped into four functional clusters: GTPase regulator activity (enrichment score = 1.17), protein complexes assembly (enrichment score = 0.76), regulation of transcription (enrichment score = 0.45), and nucleotides and ribonucleotides binding (enrichment score = 0.13), but none are significant. A total of 24 genes are within the seven overlapping regions across the East (KEASZ and UGN) and West African (NGR) populations (Supplementary Table 13). These genes are grouped into a single cluster associated with ion binding (enrichment score = 0.14).

A total of 11,915 SNPs in 148 genes and 484 indels in 96 genes are identified within the 35 overlapping genome-wide SNP and *Hp* candidate regions. These variants are either located on the coding region (missense, synonymous SNPs, and frameshift indels), or non-coding regions (intronic, splice regions, and 3′ and 5′ UTR SNPs and indels; Supplementary Table 14). A total of 261 SNPs in 50 genes and eight indels in seven genes have not been reported yet in the dbSNP database “novel variants.” Among all the variants, 88 SNPs in 49 genes (one novel) are missense, 50 SNPs in 37 genes (none novel) are on splice regions, two indels in two genes (none novel) are frameshift and two indels in two genes (none novel) are on splice regions (Supplementary Table 14).

Seventy-five missense SNPs in 44 genes were also identified in the KEASZ exome data spanning the overlapping regions. Twenty-three of these SNPs, in 13 genes, were not present in African taurine (Muturu and N'Dama), they are likely of zebu origin in KEASZ. Whilst, 15 missense SNPs in 12 genes were also identified in the two African taurine cattle populations examined and therefore they may be of taurine origin. Among the 50 splice regions SNPs, 44 in 32 genes are identified in the KEASZ exome data, in which seven SNPs in six genes may be of zebu origin and ten SNPs in nine genes may be of taurine origin (Supplementary Table 15). Only a single indel classified as frameshift and splice region indel has been identified in the KEASZ exome data. This indel has also been found in the genome of N'Dama and Muturu cattle and may be of taurine origin (Supplementary Table 15).

Twelve genes within the overlapping genome-wide SNP and *Hp* candidate regions were selected as examples of interesting candidates of positive selection in KEASZ following their biological roles (Table 8). These genes have functional roles linked to traits for adaptation to the African environment and/or reproductive fitness, e.g., immunological-related traits (e.g., disease challenges) and reproduction-related traits (e.g., fertility).

**Table 8:** Candidate genes within the KEASZ overlapping genome-wide SNP and *Hp* candidate signatures of selection regions.

**Biological role**	**Candidate genome region**	**Gene ID**	**Gene description**
Immunity	BTA 19: 40,960,001–41,450,870	*CSF3*	colony stimulating factor 3 (granulocyte)
	BTA 19: 40,960,001–41,450,870	*CCR7*	chemokine (C-C motif) receptor 7
Fertility and reproduction	BTA 12: 29,110,001–29,438,417	*RXFP2*	relaxin/insulin-like family peptide receptor 2
	BTA 19: 40,960,001–41,450,870	*RARA*	retinoic acid receptor, alpha
	BTA 7: 51,360,001–53,362,761	*SPATA24*	spermatogenesis associated 24
	BTA 19: 26,890,001–27,154,002	*SPAG7*	sperm associated antigen 7
Heat stress	BTA 2: 125,640,001–126,083,262	*DNAJC8*	dnaJ (Hsp40) homolog, subfamily C, member 8
	BTA 7: 51,360,001–53,362,761	*DNAJC18*	dnaJ (Hsp40) homolog, subfamily C, member 18
	BTA 7: 51,360,001–53,362,761	*HSPA9*	heat shock 70kDa protein 9
	BTA 19: 42,890,001–43,122,753	*HSPB9*	heat shock protein, alpha-crystallin-related, B9
Anatomical development	BTA 5: 48,610,001–49,021,113	*LEMD3*	inner nuclear membrane protein
	BTA 7: 33,100,001–33,293,306	*LOX*	lysyl oxidase
	BTA 12: 29,110,001–29,438,417	*RXFP2*	relaxin/insulin-like family peptide receptor 2

Polymorphisms were identified in all these genes with the exception of the *HSPB9* gene, which was found monomorphic. A total of 668 SNPs and 32 indels were detected (Supplementary Table 16). In particular, four missense variants on three genes, one splice region SNP on one gene and one frameshift indel on one gene were identified (Table 9), in which all have been reported previously in the dbSNP database (Sherry et al., 2001). Both of the two missense variants in *RXFP2* are considered as of probable zebu origin. Whilst the splice region SNP on *SPATA24* is considered as of probable taurine origin (Table 9, Supplementary Table 15).

**Table 9:** Missense, splice region SNPs, and frameshift indels within KEASZ candidate genes under positive selection.

**Variant location**	**Gene**	**Variant type**	**Amino acid change**	Alternative allele frequency^*^	Biological effect^**^	**Origin**
BTA 5: 48,781,557	*LEMD3*	Frameshift	L 679 FX	F = 0	Undefined	Undefined
BTA 5: 48,781,846	*LEMD3*	Missense	T 665 I	A = 0.2	Probably damaging	Undefined
BTA 7: 52,298,800	*SPATA24*	Splice region SNP	–	G = 1	Undefined	Taurine
BTA 12: 29,243,223	*RXFP2*	Missense	C 459 G	C = 0.4	Probably damaging	Zebu
BTA 12: 29,280,777	*RXFP2*	Missense	N 19 S	C = 65%	Benign	Zebu
BTA 19: 27,072,057	*SPAG7*	Missense	R 144 Q	A = 0	Benign	Undefined

* *Based on the 10 KEASZ exome sequences*,

** *Based on PolyPhen-2 online tool (Adzhubei et al., 2010)*.

### Estimation of excess-deficiency in Asian zebu ancestry

LAMP software 2.4 (Sankararaman et al., 2008) estimated the mean Asian zebu ancestry proportion for all autosomal SNPs in KEASZ to be 0.76 (SD = 0.14). Based on this estimation, the mean ΔAZ for autosomal SNPs is 0 (SD = 0.14). The majority of the candidate sweep regions show high zebu ancestry proportion, but similar to the mean Asian zebu ancestry proportion (within one SD from the mean). For the 32 East African candidate regions (Table 3), eight regions reveal substantial ΔAZ, a single region shows deficiency and seven show excesses (more than ± one SD from the mean ΔAZ). Moreover, six East and West African candidate regions (Table 3) demonstrate substantial ΔAZ. Two of these regions show deficiencies and four show excesses of Asian zebu ancestry. When the genome-wide SNP and *Hp* analyses overlapping KEASZ candidate regions were considered, 13 regions demonstrate substantial ΔAZ. One region, which is shared between East and West African cattle, show deficiency in Asian zebu ancestry. Whilst 12 regions show excess in Asian zebu ancestry, in which six are specific to East African cattle and one is shared between East and West African cattle (Table 7).

## Discussion

In this study, we unravel the autosomal zebu × taurine admixed genome structure of African indigenous cattle populations from the eastern (Kenya and Uganda) and western (Nigeria) part of the African continent using genome-wide high density SNP data. Also, for the first time, both genome-wide SNP and full genome sequence data have been utilized to identify candidate signatures of positive selection in the genome of an African cattle, the EASZ from Kenya, based on meta-analysis of selection signals (*meta-SS*) and pooled heterozygosity (*Hp*) analysis. These regions were then further characterized to identify candidate causative variants and to assess their probable zebu or African taurine origins.

### Genetic structure of East and West African Cattle populations

Archaeological and genetic evidences so far indicate that the history of African zebu cattle started with the introgression of Asian zebu to the native African taurine populations ~4,000 and 1,300 years ago (Epstein, 1971; Hanotte et al., 2002). The coordinates of the African cattle samples (KEASZ, UGN, and NGR) in the PCA plots (Figures 1A,B) indicate zebu × taurine admixture level in their genome. This zebu introgression appears even across animals within population on the autosomes of KEASZ and also in West African cattle from Nigeria as well as in Ankole and Karamojong zebu cattle from Uganda (Figure 2). At the contrary, uneven European taurine introgression, likely of recent origin following ongoing crossbreeding of indigenous African cattle populations with exotic cattle breeds to improve their productivity (Mwai et al., 2015), is observed in the genome of Nganda and Serere zebu cattle from Uganda (Figure 2).

Interestingly, we also observe positive significant correlation in Asian zebu ancestry autosomal proportion between the KEASZ and the other East and West African cattle populations examined. This is in agreement with the known history of zebu cattle on the continent (Hanotte et al., 2002) and it supports a common ancestry for the African cattle examined here. Our results also support an East African origin for the West African zebu cattle as previously showed in Hanotte et al. (2002) with migration of admixed zebu × taurine cattle populations.

### Candidate genomics regions under positive selection

This study is the first to our knowledge that exhaustively investigated the genome of an indigenous African cattle population for signatures of positive selection using both high density genome-wide SNP and full genome sequence information. The outputs of these analyses represent a follow up of our previous identifications of signatures of selection on KEASZ genome using the lower density Illumina BovineSNP50 BeadChip v.1 (Bahbahani et al., 2015). Here, we have validated 14 candidate regions out of 24 regions previously identified to be targeted by positive selection in KEASZ (Supplementary Table 17; Bahbahani et al., 2015). The remaining 10 regions might have been false positives. Indeed, in our previous study, we used a genomic tool characterized by high European taurine ascertainment bias and low genome coverage. This issue has been addressed in this study by using high-density HD SNP array and full genome sequence data. Moreover, we previously selected candidate regions based on only two SNPs above the significant threshold. Here we have been using much more stringent criteria with a minimum of five SNPs above the threshold to define a candidate region under positive selection (see Materials and Methods Section).

We also used a different strategy to increase the power of detecting genomic signatures of positive selection. First, instead of analyzing regions defined by each genome-wide SNP analyses (*Rsb, iHS*, and Δ*AF*) separately, a composite statistical approach “*meta-SS* analysis” was conducted at the autosomal level as in Utsunomiya et al. (2013) to combine the SNP-specific *P*-values for each test into a single index.

Simulation data have shown that combining the signals from different tests into a single statistic increase the power of defining genomic regions under selection (Grossman et al., 2010). Most importantly, coalescent simulations and accurate calibrated demographic models, which are lacking in African cattle, are not required by *meta-SS* in comparison to the original Composite of Multiple Signals (CMS) method proposed by Grossman et al. (2010). Secondly, the reference cattle populations were pooled into a single population as in Bahbahani et al. (2015). As suggested by Gautier and Naves (2011), this pooling approach increases the haplotype diversity in the reference populations and it breakdown population-specific linkage disequilibrium (LD) which may result from genetic drift. Thirdly, information from both genome-wide SNP genotypes and full genome sequence were used to adequately cover the KEASZ genome, as well as, to address any breed ascertainment bias associated with the commercially available SNP arrays (Matukumalli et al., 2009). Overlapping candidate regions from these two approaches further support the identification of the candidate regions.

### The candidate regions: a result of positive selection or genetic drift?

Both selection and genetic drift may have shaped the genome of the KEASZ and the other populations examined here. Distinction between the two is difficult, and fixation or near fixation of allele through genetic drift may lead to false positive candidate regions for signatures of positive selection (Qanbari and Simianer, 2014). Comparison of the results between different cattle populations, the unraveling of the zebu or taurine origin of the selected regions and the function of genes within selected regions may here provide further information.

In this study, we observe a small number of candidate regions for positive selection shared between KEASZ HD SNP and/or genome sequencing information and the East and West African cattle population examined here (Tables 3, 7). While it may be argued that these overlapping candidate regions for positive selection are a consequence of common genetic backgrounds, the low number of shared candidate regions as well as the identification of the same regions in tropical-adapted cattle populations with different population histories, e.g., Creole cattle from Guadeloupe (Gautier and Naves, 2011), zebu × taurine admixed cattle from West Africa (Gautier et al., 2009; Flori et al., 2014; Xu et al., 2015), Brahman (Ramey et al., 2013; Xu et al., 2015) and Gir (Liao et al., 2013; Perez O'Brien et al., 2014) cattle (Tables 3, 7, and Supplementary Table 11) strongly supports the role of selection rather genetic drift for these shared regions. It underlines the importance that these genome regions may play a role in the adaptive traits of African tropical-adapted admixed cattle.

Examining the probable ancestral origin of the candidate regions reveals a subset of candidate regions with substantial ΔAZ (Tables 3, 7). Most of these regions show excesses of Asian zebu ancestry, e.g., BTA 7: 51.4–53.4 Mb and BTA 13: 47.5–48.1 Mb, indicating that the indicine haplotypes are more likely to be under selection in the African admixed cattle populations than the taurine. This is perhaps not surprising considering the predominant zebu genomic background in EASZ (Mbole-Kariuki et al., 2014). However, we also observed candidate regions showing substantial excess of African taurine ancestry, e.g., BTA 3: 76.1–76.4 Mb and BTA 19: 3.3–3.8 Mb (Table 3). These are present in chromosomes with overall low level of African taurine ancestry adding further support for selective pressure rather than genetic drift for their presence (Table 2).

### Biological functions of the genes present in signatures of selection regions in EASZ

Examining the potential biological functions under positive selection reveal several different significantly enriched biological pathways likely under selection in KEASZ given their importance for a cattle population living in a challenging tropical environment (e.g., bovine adaptive and innate immunity, response to hormone stimuli, intermediate filaments, and keratins pathways). Additionally, genes and QTL related to regulation of bovine immunity, fertility and reproduction, anatomical development, and heat stress have also been found within the identified KEASZ candidate regions (Supplementary Tables 9, 12, 18).

Indeed, innate and adaptive immune genes may be expected to be primary targets of selection in African cattle that are exposed to a diversity of pathogens and associated physiological stresses in their surrounding environment, e.g., endoparasites, haemoparasites, and bacteria (de Clare Bronsvoort et al., 2013; Murray et al., 2013; Thumbi et al., 2014). Examples of candidate genes related to this category are: C-C chemokine receptor type 7 precursor (*CCR7*) and granulocyte macrophage-colony stimulating factor (*CSF3*). Upon binding to two chemoattractants: CCL19 and CCL21, *CCR7* is involved in maturating dendritic cells and hence activate T lymphocytes (Marsland et al., 2005; Forster et al., 2008). This receptor has also demonstrated a role in regulating innate immunity by attracting macrophages to sites of infection (van Zwam et al., 2010). The multifunctional cytokine (CSF3) acts as a positive regulator for macrophages to induce their antimicrobial effects (Grabstein et al., 1986; Tarr, 1996). Interestingly, several trypanotolerance QTL, identified by Hanotte et al. (2003), overlap with the identified KEASZ candidate regions (Supplementary Table 19). These QTL might indicate an undocumented level of trypanotolerance in KEASZ, as it has already been shown in other East African cattle populations (e.g., Orma Boran, Sheko and Mursi cattle; Dolan, 1987; Mwangi et al., 1993; Bahbahani and Hanotte, 2015).

Our results also show that besides tolerance to disease challenges, fertility and reproduction traits have been also selected in the African zebu × taurine admixed cattle. Examples of these candidate genes are: the retinoic acid receptor α subunit (*RARA*), relaxin/insulin-like family peptide receptor 2 (*RXFP2*), spermatogenesis associated 24 (*SPATA24*), and sperm associated antigen 7 (*SPAG7*). The retinoic acid receptor, which is expressed in sertoli cells in the seminiferous tubules, plays a role in maintaining retinoic acid signal to induce spermatogonia differentiation (Wolgemuth and Chung, 2007). *RXFP2*, which has been found to be under selection in admixed Creole cattle (Gautier and Naves, 2011) and Gir cattle (Liao et al., 2013), plays a role in testicular descent development (Gorlov et al., 2002; Agoulnik, 2007; Park et al., 2008; Feng et al., 2009). Candidate genes such as spermatogenesis-associated 24 (*SPATA24*) and sperm-associated antigen 7 (*SPAG7*) can also be classified into the cattle fertility and sexual reproduction category due to their role in spermatogenesis. Such signals may be the legacy of selection for fertility in hybrid populations between two cattle lineages (zebu and taurine), with a common ancestry perhaps as old as half a million years ago (MacHugh et al., 1997). This requires further investigation. QTL related to bovine reproduction and fertility, e.g., sperm motility and calving ease, are also target of positive selection in KEASZ.

An important gene category identified within the candidate regions is the heat stress category. Genes and QTL within this category might be targeted by natural selection to adapt to the tropical environmental condition (Hansen, 2004). Two heat shock protein genes (*HSPA9* and *HSPB9*) and two members of the DnaJ family (*DNAJC8* and *DNAJC18*) are mapped within three of the identified candidate regions (Table 8). Heat shock proteins have critical roles in maintaining protein folding under heat stress (Parsell and Lindquist, 1994; Coleman et al., 1995). The members of DnaJ family act as cofactors for other heat shock proteins (Hsp70) to maintain protein folding (Kampinga and Craig, 2010).

Genes and QTL related to anatomical development were also identified within the KEASZ candidate regions (e.g., *LEMD3, LOX*, and *RXFP2*). These genes are important to maintain optimum growth and development. *LEMD3* and *LOX* are associated with the development of different organs, such as heart (*LEMD3*), lung and blood vessels (*LOX*) (Maki et al., 2005; Ishimura et al., 2008). The candidate region harboring *LEMD3* has also been found to be under selection in Brahman cattle (Ramey et al., 2013). The role of *RXFP2 in* testicular descent development (Gorlov et al., 2002; Agoulnik, 2007; Park et al., 2008; Feng et al., 2009) can classify this gene into the anatomical development, fertility and reproduction as well as the heat tolerance categories. This gene has also been associated with the horn phenotype in sheep (Johnston et al., 2011; Kijas et al., 2012) and a study by Johnston et al. (2013) has demonstrated an association between variants of this gene and reproductive success and survival rate in Soay sheep from St. Kilda.

In addition, various production traits QTL, e.g., marbling score, milk fat percentage, and milk fat yield have also been found within the KEASZ candidate regions. Given that several of the identified KEASZ candidate regions overlap with regions under positive selection in commercial dairy and beef cattle breeds (Tables 3, 7, Supplementary Table S11; Larkin et al., 2012; Kemper et al., 2014; Qanbari et al., 2014), this may illustrate possible human selection for some production traits, e.g., milk yield, has taken place in KEASZ at least in the past.

In parallel to candidate regions harboring genes, the 39 gene desert candidate regions on KEASZ are targets of further research to define their biological roles. Although these regions do not contain any of the bovine transcription factors binding sites identified by Bickhart and Liu (2013), they may still harbor unannotated regulatory elements and/or genes targeted by positive selection. These regions may also be transcribed to generate long non-coding RNA transcripts (≥200 nucleotides), which could be further validated by RNA sequencing. This type of RNA molecules has critical roles in regulating the expression of neighboring genes at transcriptional and post-transcriptional levels (Mercer et al., 2009; Wang and Chang, 2011).

### Putative causative variants in candidate regions

Several genomic variants, in which some are unique so far to KEASZ, have been identified within the KEASZ overlapping genome-wide SNP and *Hp* candidate regions. A subset of these variants, such as missense SNPs, framshift indels, and splice region SNPs/indels, can be considered as primary target of positive selection due to their functional roles on the corresponding genes. Interestingly, within the selected candidate genes four missense SNPs, a splice region SNP and a frameshift indel have been identified. These variants need to be confirmed in larger sample size of tropical-adapted (e.g., EASZ) and non-tropical-adapted (e.g., European taurine) cattle to further support their role as causative variants under selection. Two of the missense variants are predicted to show non-benign effects on their genes calling for their effect to be validated, e.g., through gene editing approaches (Carlson et al., 2012; Wang et al., 2013). Moreover, variants on the other genes within the candidate regions or in non-coding regions following their possible roles in regulating gene expression may also be targets of selection.

Based on available sequences of KEASZ and African taurine cattle (N'Dama and Muturu), we were able to infer the possible ancestral origin of some of these functional variants (Asian zebu or African taurine origin) indicating the role of the zebu × taurine admixture as a selective force shaping the genome of KEASZ. However, this origin assignment was only partially successful due to the low number of sequenced samples and the unavailability of a full indicine *de novo* reference genome.

## Conclusion

We reported here for the first time extensive exploring for candidate regions and putative causative variants under selection on the genome of an indigenous African cattle (East African shorthorn zebu) using both genome-wide SNP data and full genome sequence data. The possible ancestral origins of some of these variants have been inferred using exome data of EASZ from Kenya and full genome sequence of African taurine cattle. In this study we have defined three selective forces on the KEASZ genome; the external environmental pressures, the internal admixed genome pressure and possible ancient human selection for production traits. Our results can be considered as the first milestone in conserving the adaptive genetic resources of the indigenous African cattle and to further improve their breeding strategy. This will enhance the productivity of the indigenous African cattle populations and at the same time retain their African environment adaptability. Although in this study we have used two genomic tools and cattle populations from East and West of Africa, these results need to be further validated in larger number of cattle populations with different ancestries and environments.

## Data accessibility

The pooled full genome sequence and the 10 exome sequences of the East African shorthorn zebu, in addition to the Muturu full genome sequences, are publicly available from GenBank with the Bioproject accession number PRJNA386202. All identified SNPs are deposited in dbSNP under handel (UON_LAB_A100). The high density SNP genotyping data available from the Dryad Digital Repository: https://doi.org/10.5061/dryad.38jp6.

## Ethics statement

Standard techniques were used to collect blood. The procedure was reviewed and approved by the University of Edinburgh Ethics Committee (reference number OS 03-06) and also by the Institute Animal Care and Use Committee of the International Livestock Research Institute, Nairobi.

## Author contributions

HB and OH conceived, designed the experiment. HB and OH performed the experiment. HB, AT, and FA analysed the data. MB and DW helped in the bioinformatic analysis. TS, MW, HH, ON, AT, GA, CM, MM, and CV contributed in data. HB and OH wrote the manuscript. All authors have agreed on the contents of the manuscript.

### Conflict of interest statement

The authors declare that the research was conducted in the absence of any commercial or financial relationships that could be construed as a potential conflict of interest. The reviewer MB and handling Editor declared their shared affiliation, and the handling Editor states that the process nevertheless met the standards of a fair and objective review.

## Abbreviations

KEASZ: East African Shorthorn Zebu from Kenya
UGN: Uganda
NGR: Nigeria
AO: Ankole
KR: Karamojong zebu
NG: Nganda
ZS: Serere zebu
AG: Adamawa Gudali
AZ: Azawak
BJ: Bunaji
OR: Red bororo
SO: Sokoto Gudali
WD: Wadara
YK: Yakanaji.
