# Supplementary material for: Signatures of Selection for Environmental Adaptation and Zebu × Taurine Hybrid Fitness in East African Shorthorn Zebu
Source: Front Genet. 2017 Jun 8;8:68. doi: 10.3389/fgene.2017.00068 (PMC5462927; doi:10.3389/fgene.2017.00068)
Supplement: Supplementary file 10 [file DataSheet1.docx]

Supplementary Material

**Signature of selection for environmental adaptation and zebu x taurine hybrid fitness in East African Shorthorn Zebu**

**Hussain Bahbahani^*^,Abdulfatai Tiijani, Christopher Mukasa, David Wragg, Faisal Almathen, Oyekanmi Nash, Gerald N. Akpa, Mary Mbole-Kariuki, Sunir Malla, Mark Woolhouse, Tad Sonstegard, Curtis V. Tassell, Martin Blythe, Heather Huson and Olivier Hanotte**

**Correspondence:**

Hussain Bahbahani

h.bahbahani@hotmail.com

hussain.bahbahani@ku.edu.kw

**
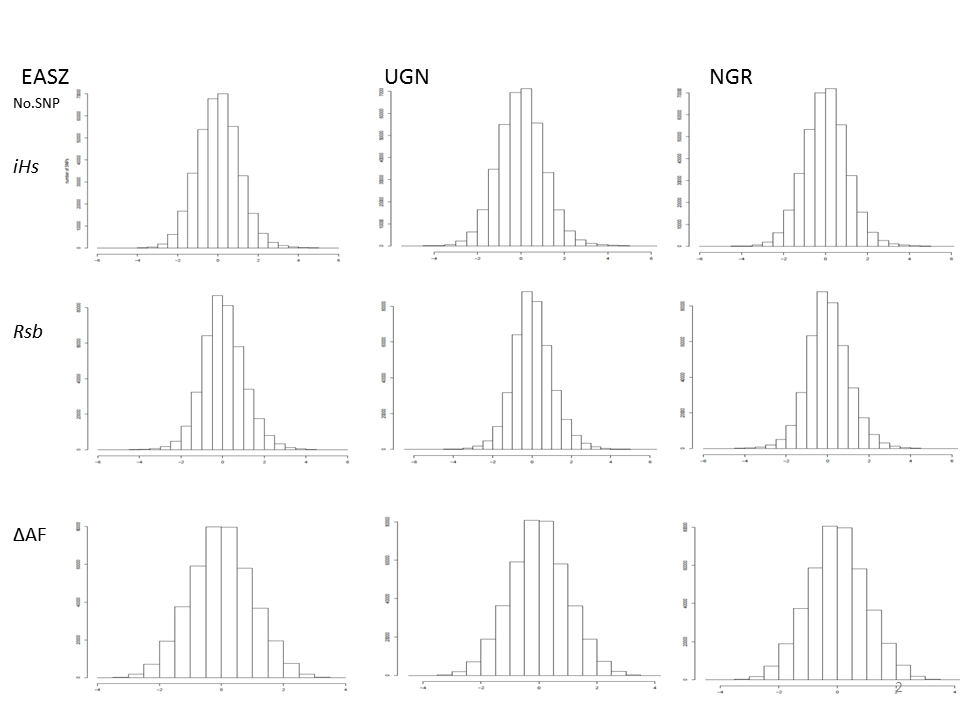
**

**KEASZ**

**Supplementary Figure 1**: Histogram plots of *Rsb*, *iHS* and ΔAF standardized values performed on the autosomal SNPs.


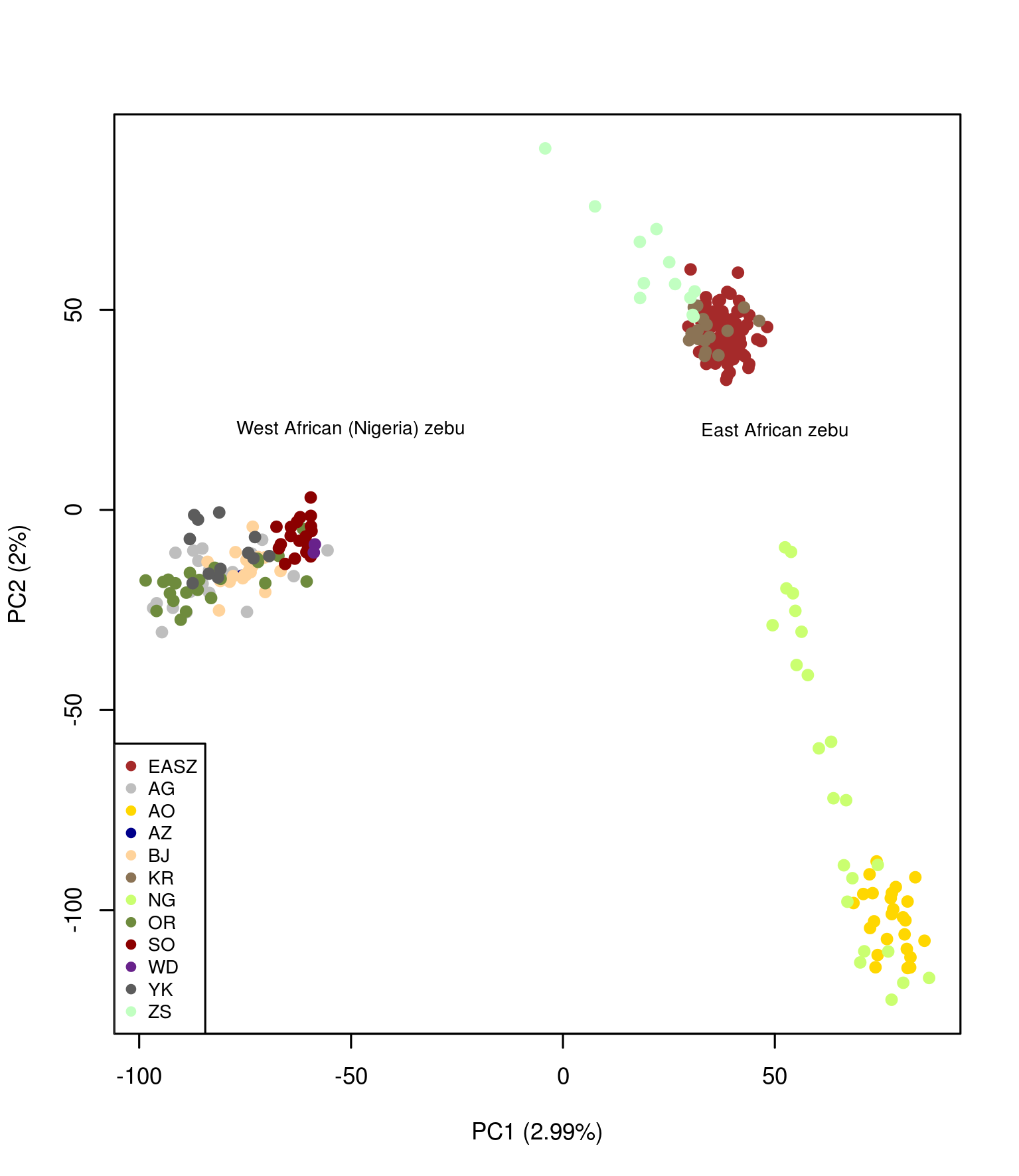


East African cattle (KEASZ and UGN)

**Supplementary Figure 2**: Plot of the highest two principle components resulted by analyzing autosomal SNPs in KEASZ (East African shorthorn zebu from western Kenya), East African Shorthorn Zebu, Sanga and Zenge cattle from Uganda (AO: Ankole. KR: Karamojong zebu. NG: Nganda. ZS: Serere zebu) and West African zebu cattle from Nigeria (AG: Adamawa Gudali. AZ: Azawak. BJ: Bunaji. OR: Red bororo. SO: Sokoto Gudali. WD: Wadara. YK: Yakanaji)

**
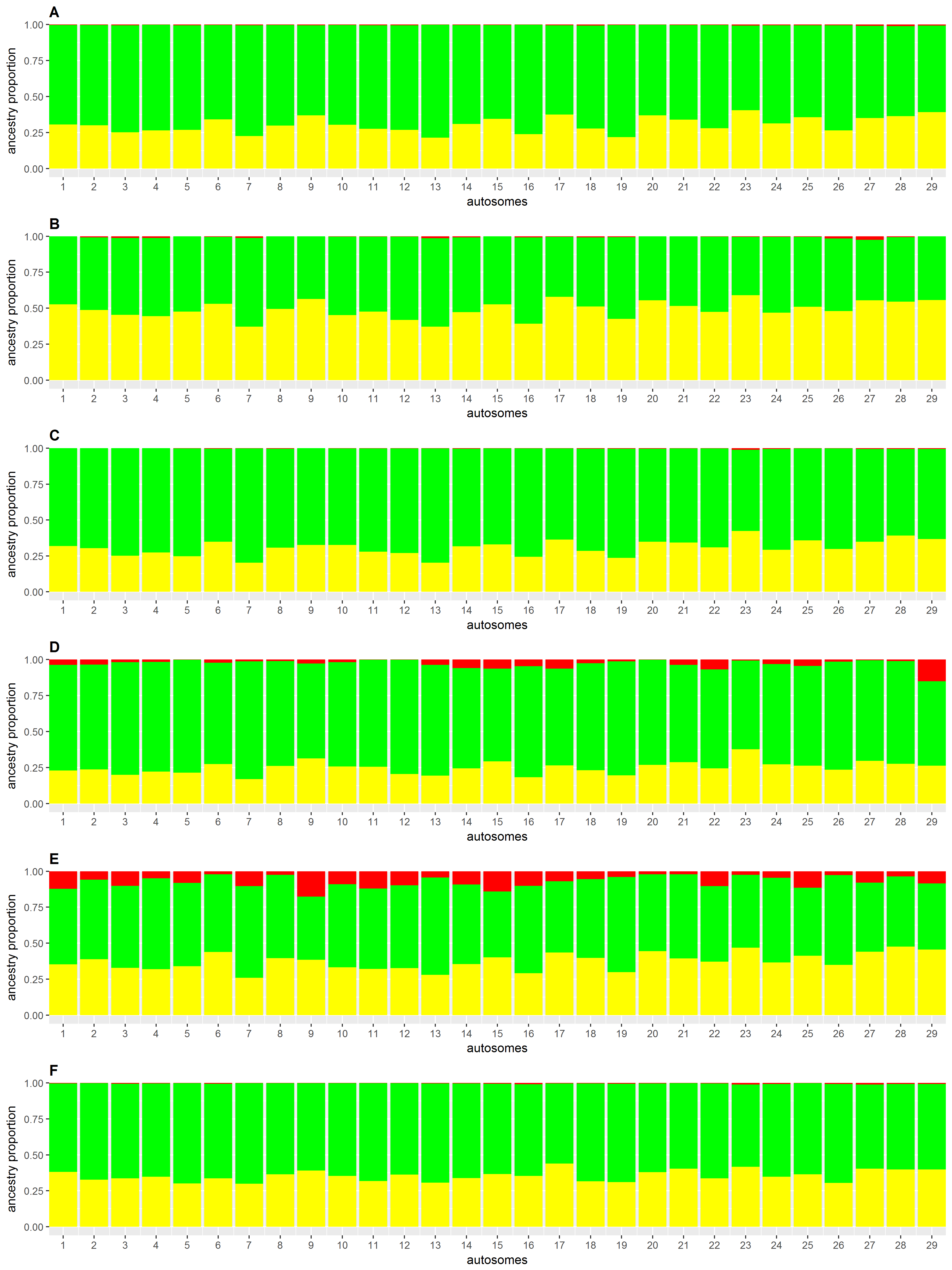
**

**Supplementary Figure 3:** Variation in Asian zebu (green), African taurine (yellow) and European taurine (red) ancestry proportions across the autosomes of (A) East African shorthorn zebu from western Kenya (B) Ankole, (C) Karamojong zebu, (D) Serere zebu, (E) Nganda and (F) West African zebu cattle from Nigeria.


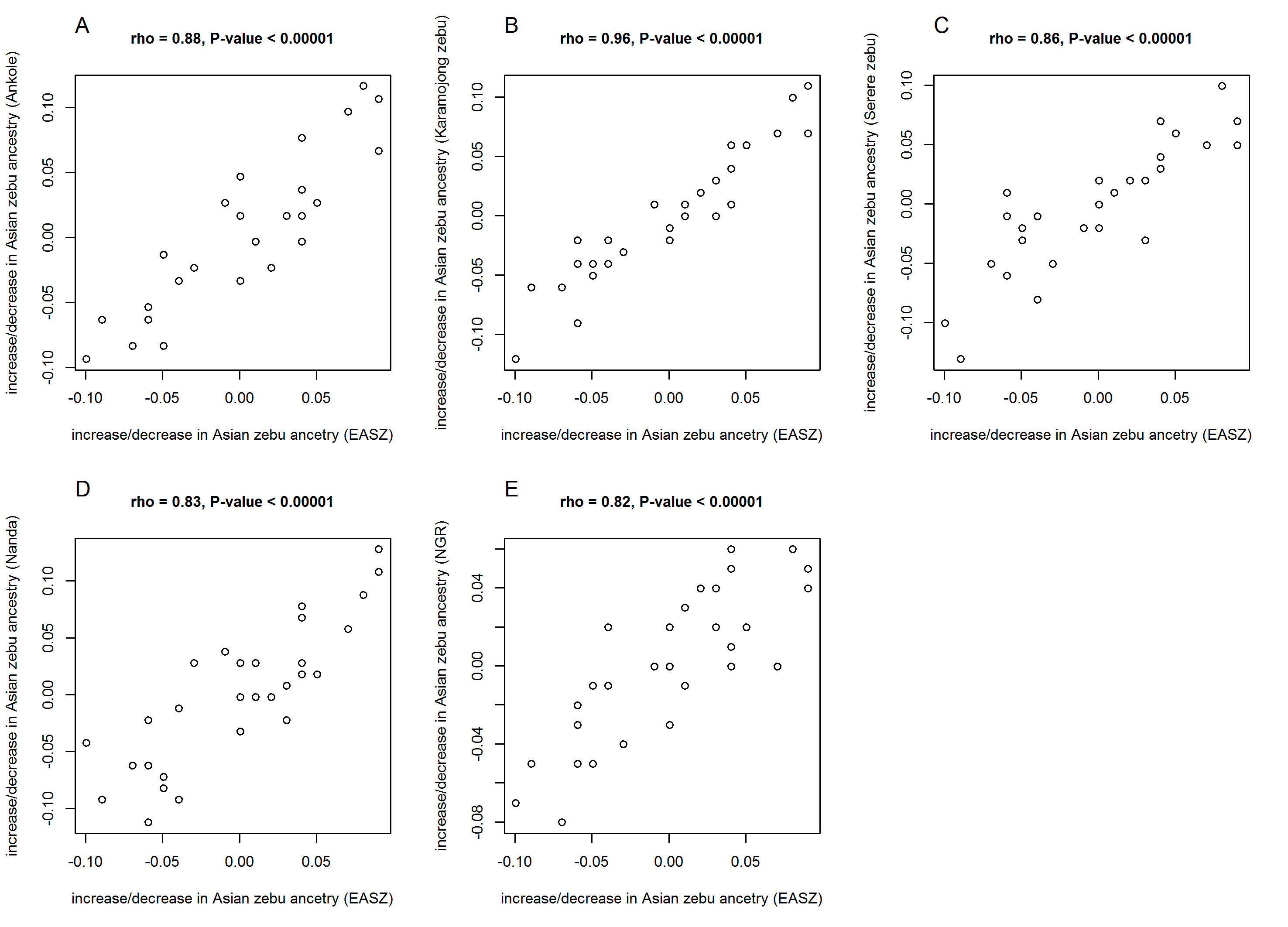


**Supplementary Figure 4:** Spearman's rank correlation between increase/decrease in Asian zebu ancestry of KEASZ autosomes and (A) Ankole autosomes, (B) Karamojong zebu autosomes, (C) Serere zebu autosomes, (D) Nganda autosomes and (E) West African zebu cattle from Nigeria (NGR) autosomes.


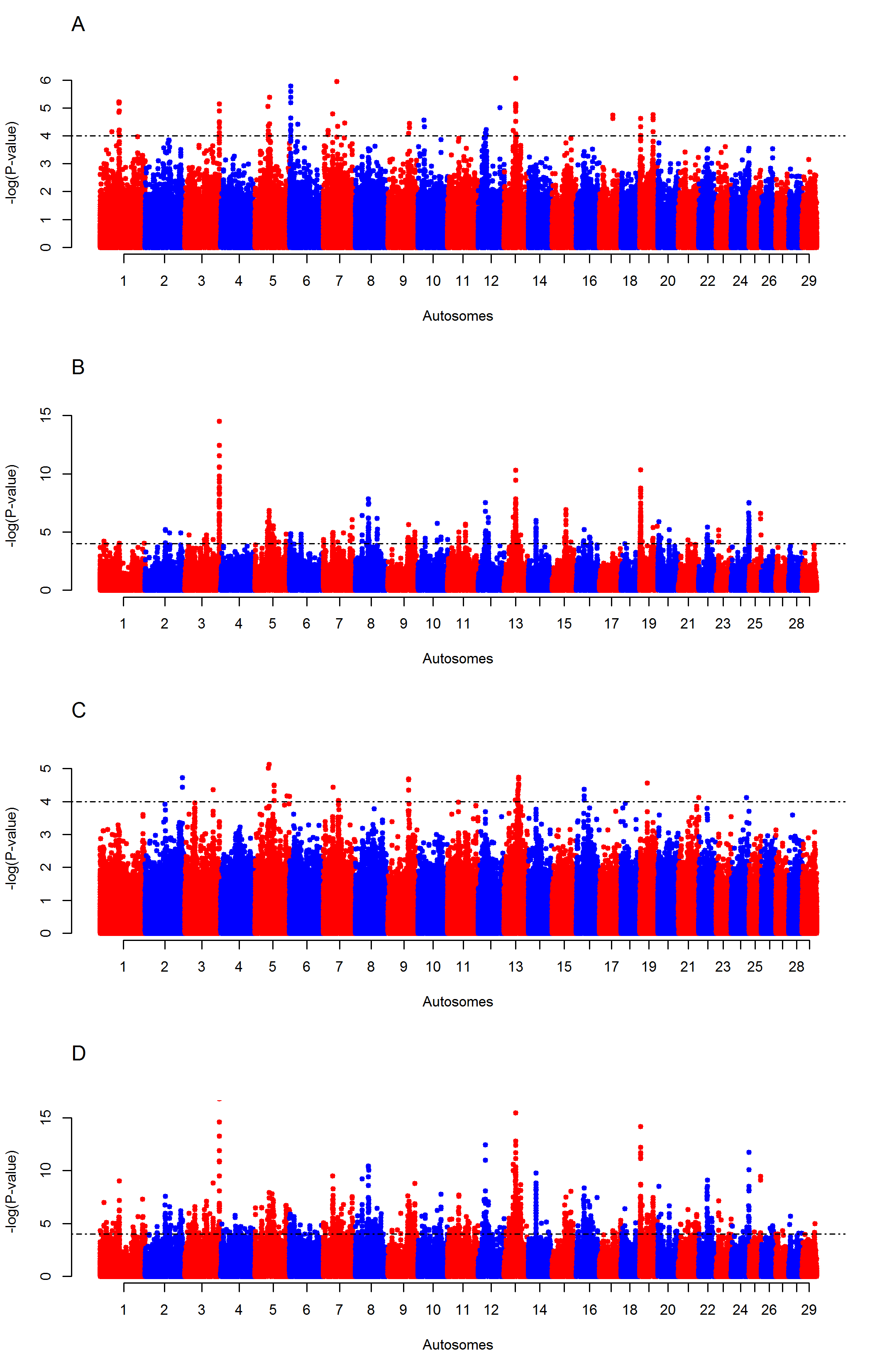


**Supplementary Figure 5**: Manhattan plots for the autosomal **(A)** *iHS* on East African cattle populations from Uganda (UGN), **(B)** *Rsb*, **(C)** ΔAF and **(D)** *meta-SS* analyses between UGN and combined reference populations (Holstein-Friesian, Jersey, N’Dama, Muturu, Nelore and Gir). Threshold set as – log_10_ *P*-value = 4.


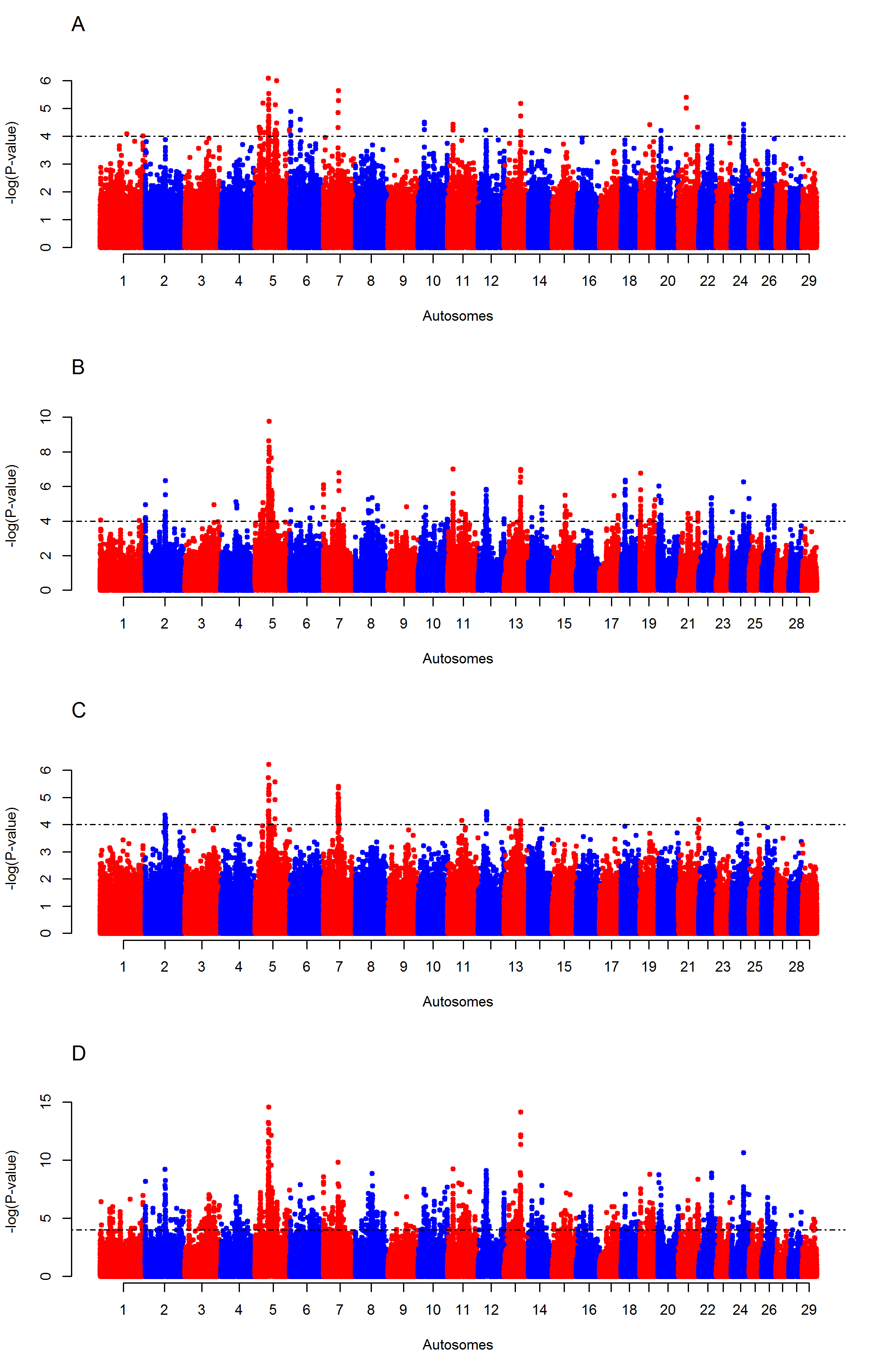


**Supplementary Figure 6**: Manhattan plots for the autosomal **(A)** *iHS* on zebu cattle populations from Nigeria (NGR), **(B)** *Rsb*, **(C)** Δ*AF* and **(D)** *meta-SS* analyses between NGR and combined reference populations (Holstein-Friesian, Jersey, N’Dama, Muturu, Nelore and Gir). Threshold set as – log_10_ *P*-value = 4.


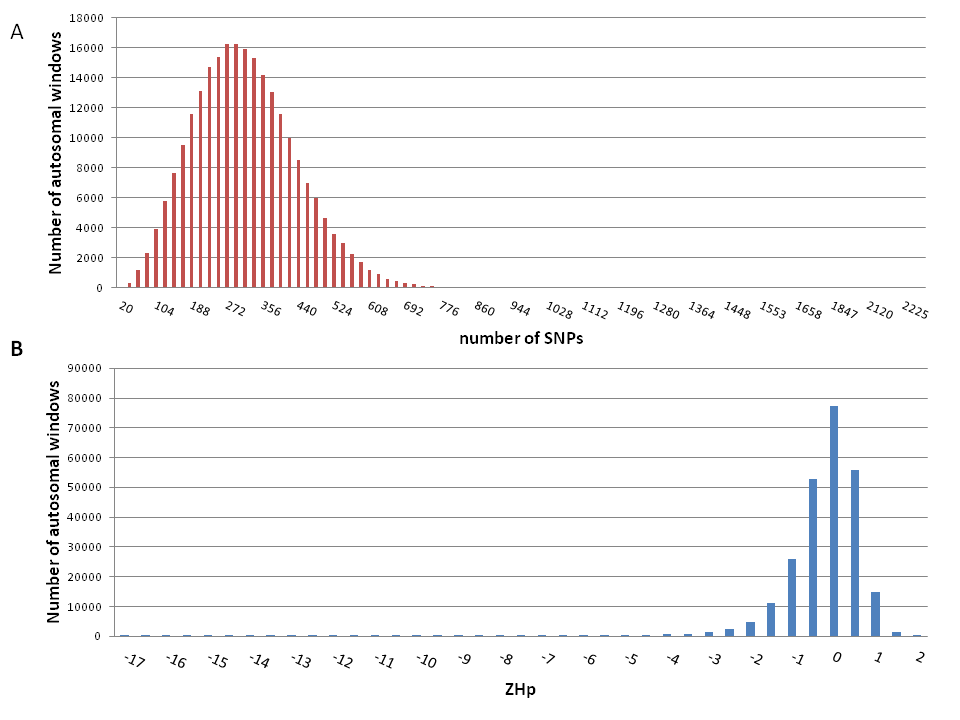


**Supplementary Figure 7**: Distribution of SNPs in the 100 kb autosomal windows on KEASZ.

**Supplementary Table 1:** The EASZ samples from western Kenya in the two full genome sequence pools, and the ten exome sequence samples. Their sublocations and sex.

| **Full genome sequence** |  |  |
| --- | --- | --- |
| **Sample ID (DEDG number)** | **Sublocation** | **Sex** |
| **354** | Namboboto | male |
| **90** | Bukati | male |
| **500** | Bujwanga | female |
| **341** | Kidera | female |
| **364** | Otimong | female |
| **352** | Magombe East | male |
| **378** | Bumala | male |
| **70** | Mabusi | male |
| **194** | Kokare | female |
| **18** | Bujwanga | female |
| **Exome sequence** |  |  |
| **Sample ID (DEDG number)** | **Sublocation** | **Sex** |
| **289** | Kidera | female |
| **1693** | Otimong | male |
| **524** | MagombeEast | female |
| **915** | SimurEast | female |
| **1148** | Bujwanga | female |
| **2025** | Bujwanga | male |
| **2063** | OjwandoB | male |
| **1401** | OjwandoB | male |
| **923** | Ikonzo | female |
| **2183** | Ikonzo | male |

**Supplementary Table 2:** A summary of the exome sequence reads mapped to the UMD3.1 bovine reference genome for each sequenced EASZ sample from western Kenya.

| **Sample ID (DEDG number)** | **289** | **524** | **915** | **923** | **1148** | **1401** | **1693** | **2025** | **2063** | **2183** |
| --- | --- | --- | --- | --- | --- | --- | --- | --- | --- | --- |
| **Total Raw Reads** | 60,521,890 | 61,886,159 | 76,115,987 | 71,041,457 | 54,406,488 | 70,583,687 | 75,825,024 | 97,926,144 | 56,892,642 | 81,334,699 |
| **Mapped Reads (MAPQ0)** | 58,123,736 | 59,259,487 | 72,520,518 | 67,565,041 | 52,179,782 | 67,757,017 | 72,357,186 | 93,087,864 | 54,589,349 | 77,623,887 |
| **Total MAPQ30 Aligned Reads** | 52,530,808 | 53,401,659 | 65,074,820 | 60,547,213 | 47,286,573 | 61,153,497 | 65,090,879 | 84,299,461 | 49,335,902 | 69,825,532 |
| **Reads In Targets:** | 46,060,296 | 47,152,655 | 56,371,737 | 52,981,396 | 41,868,685 | 54,020,004 | 57,191,616 | 75,303,053 | 43,725,770 | 61,038,375 |
| **Reads Off Targets:** | 6,470,512 | 6,249,004 | 8,703,083 | 7,565,817 | 5,417,888 | 7,133,493 | 7,899,263 | 8,996,408 | 5,610,132 | 8,787,157 |
| **Percent of Target Bases Not Covered** | 8.58% | 8.68% | 7.77% | 7.56% | 9.23% | 8.30% | 7.90% | 6.31% | 8.83% | 7.26% |
| **Percent of Target Bases Covered** | 91.42% | 91.32% | 92.23% | 92.44% | 90.77% | 91.70% | 92.10% | 93.69% | 91.17% | 92.74% |
| **Average Depth of Target Coverage** | 52.82 | 54.09 | 64.3 | 60.72 | 47.96 | 62.11 | 65.42 | 87.17 | 50.37 | 70.02 |

**Supplementary Table 3:** A summary of the genome sequence reads mapped to the UMD3.1 bovine reference genome for each sequenced Muturu sample from Nigeria.

| **Sample ID** | **DNA sequenced (Gb)** | **Total reads** | **Alignment rate** | **Mean depth** | **Genome coverage (%)** |
| --- | --- | --- | --- | --- | --- |
| **MT001** | 30.063 | 188,884,710 | 0.99 | 8.76 | 98.4 |
| **MT002** | 32.675 | 218,811,829 | 0.99 | 9.96 | 98.8 |
| **MT003** | 36.095 | 240,635,522 | 0.99 | 10.41 | 98.5 |
| **MT004** | 35.152 | 234,425,064 | 0.99 | 9.74 | 98.3 |
| **MT005** | 32.998 | 219,989,008 | 0.99 | 8.61 | 98.4 |
| **MT006** | 31.442 | 209,619,170 | 0.99 | 6.54 | 96.8 |
| **MT007** | 31.445 | 209,630,704 | 0.99 | 6.73 | 96.8 |
| **MT008** | 29.927 | 199,312,278 | 0.99 | 7.48 | 97.6 |
| **MT009** | 33.051 | 220,336,786 | 0.99 | 8.6 | 97.7 |
| **MT010** | 31.331 | 208,872,384 | 0.99 | 6.45 | 97.4 |

**Supplementary Table S4 is published separately - see the supplementary material files**

**Supplementary Table 5:** Candidate regions from each genome-wide SNP analysis (*iHS*, *Rsb*, Δ*AF* and *meta-SS*) performed on KEASZ with the combined reference populations (Holstein-Friesian, Jersey, N’Dama, Muturu, Nelore and Gir).

| **iHS** | **Rsb** | **∆AF** | **meta-SS** |
| --- | --- | --- | --- |
| BTA 7:52824870-52929978 | BTA 3:120604441-121240490 | BTA 5:48603538-49209163 | BTA 1:21156402-22526511 |
|  | BTA 5:43269121-44220056 | BTA 7:52183599-52265269 | BTA 1:54859494-55507566 |
|  | BTA 5:48585608-49161124 | BTA 13:47508061-48142997 | BTA 1:149241884-149992523 |
|  | BTA 5:62467698-62644905 | BTA 13:48796718-48911554 | BTA 1:150577829-151624225 |
|  | BTA 7:32972540-33427191 | BTA 13:49433476-49762965 | BTA 2:70314631-71161113 |
|  | BTA 8:23344221-23663852 | BTA 13:50524278-50890135 | BTA 2:125159084-125994861 |
|  | BTA 8:75877341-76036999 |  | BTA 2:129113592-129923930 |
|  | BTA 9:70621641-70687691 |  | BTA 3:12494224-12881786 |
|  | BTA 11:62482731-62765277 |  | BTA 3:34007860-34727876 |
|  | BTA 12:35689908-35743087 |  | BTA 3:76084701-76781970 |
|  | BTA 13:40486503-40909800 |  | BTA 3:84969287-85118197 |
|  | BTA 14:28205941-28430215 |  | BTA 3:98862402-99422213 |
|  | BTA 16:24578091-25533972 |  | BTA 3:117829869-118619968 |
|  | BTA 19:2568979-2765065 |  | BTA 3:119780513-121238836 |
|  | BTA 19:40668949-42066750 |  | BTA 4:31365784-31765553 |
|  | BTA 19:46361364-46602836 |  | BTA 4:63641280-63815686 |
|  | BTA 22:45134706-46239607 |  | BTA 4:66591996-67082312 |
|  | BTA 24:61972128-62488062 |  | BTA 5:23652016-24473786 |
|  | BTA 27:4653655-4975928 |  | BTA 5:43230619-44574214 |
|  |  |  | BTA 5:48477903-49268610 |
|  |  |  | BTA 5:56651062-57515653 |
|  |  |  | BTA 5:58604207-58841085 |
|  |  |  | BTA 5:62272683-62659987 |
|  |  |  | BTA 5:109303999-110096347 |
|  |  |  | BTA 7:31748136-33875610 |
|  |  |  | BTA 7:50281923-50809190 |
|  |  |  | BTA 7:52183599-53001042 |
|  |  |  | BTA 7:61232987-61658196 |
|  |  |  | BTA 7:62415406-63117931 |
|  |  |  | BTA 7:65078295-65614779 |
|  |  |  | BTA 7:72421285-72514475 |
|  |  |  | BTA 7:84899824-85607137 |
|  |  |  | BTA 8:22905793-24288281 |
|  |  |  | BTA 8:44479612-44734659 |
|  |  |  | BTA 8:54926810-55060853 |
|  |  |  | BTA 8:64248747-64461984 |
|  |  |  | BTA 8:65301113-65634601 |
|  |  |  | BTA 8:70046025-70695580 |
|  |  |  | BTA 8:75162283-76369374 |
|  |  |  | BTA 9:69198185-69475040 |
|  |  |  | BTA 9:70454678-70753715 |
|  |  |  | BTA 9:73280867-74185868 |
|  |  |  | BTA 9:76289561-76853587 |
|  |  |  | BTA 9:85550547-85655401 |
|  |  |  | BTA 9:87580569-87599592 |
|  |  |  | BTA 9:94121197-95380876 |
|  |  |  | BTA 9:104535674-105668863 |
|  |  |  | BTA 10:80515703-80833218 |
|  |  |  | BTA 10:103294561-104290655 |
|  |  |  | BTA 11:38402190-39743107 |
|  |  |  | BTA 11:61877437-62548419 |
|  |  |  | BTA 11:71346519-72547901 |
|  |  |  | BTA 11:73807783-75446726 |
|  |  |  | BTA 12:20989169-21254061 |
|  |  |  | BTA 12:24843013-25658768 |
|  |  |  | BTA 12:27050192-29151436 |
|  |  |  | BTA 12:33250917-34546381 |
|  |  |  | BTA 12:35445176-36965854 |
|  |  |  | BTA 13:18130223-18421481 |
|  |  |  | BTA 13:38040277-38561478 |
|  |  |  | BTA 13:39430011-41356847 |
|  |  |  | BTA 13:42225984-43136553 |
|  |  |  | BTA 13:45350218-45772147 |
|  |  |  | BTA 13:50616630-50837529 |
|  |  |  | BTA 13:55428804-55542599 |
|  |  |  | BTA 13:58273562-58599491 |
|  |  |  | BTA 13:81265605-82376453 |
|  |  |  | BTA 14:28186226-28430215 |
|  |  |  | BTA 15:42536074-43195359 |
|  |  |  | BTA 16:24517859-25540339 |
|  |  |  | BTA 16:26979772-27160301 |
|  |  |  | BTA 16:40523561-41395850 |
|  |  |  | BTA 16:46869577-47614377 |
|  |  |  | BTA 16:50610769-50762363 |
|  |  |  | BTA 16:56620669-57041236 |
|  |  |  | BTA 18:13483509-14050131 |
|  |  |  | BTA 18:19446425-20061183 |
|  |  |  | BTA 19:2568979-2765065 |
|  |  |  | BTA 19:3337282-3823638 |
|  |  |  | BTA 19:9515063-10250080 |
|  |  |  | BTA 19:20622520-21225583 |
|  |  |  | BTA 19:26909816-27143239 |
|  |  |  | BTA 19:30826413-31463726 |
|  |  |  | BTA 19:39330233-39519992 |
|  |  |  | BTA 19:40045779-42066750 |
|  |  |  | BTA 19:43023638-43692285 |
|  |  |  | BTA 19:44788419-45414418 |
|  |  |  | BTA 19:46031543-46786391 |
|  |  |  | BTA 21:33590777-33696403 |
|  |  |  | BTA 21:60026698-60449172 |
|  |  |  | BTA 22:29533544-30366810 |
|  |  |  | BTA 22:42705202-44541377 |
|  |  |  | BTA 22:45102551-46400273 |
|  |  |  | BTA 24:61008938-62530799 |
|  |  |  | BTA 25:41769025-42283544 |
|  |  |  | BTA 27:4494286-5052515 |
|  |  |  | BTA 27:6938210-7107679 |
|  |  |  | BTA 28:4635419-5123022 |

**Supplementary Table 6:** Pearson correlation coefficients (r) between the *P*-values of the three autosomal genome-wide analyses (*iHS*, *Rsb* and Δ*AF*) performed on KEASZ, the zebu x taurine admixed cattle populations from Uganda and Nigeria.

| **KEASZ** | **Rsb** | **iHS** | **ΔAF** |
| --- | --- | --- | --- |
| **Rsb** | 1 | 0.228 | 0.098 |
| **iHS** | 0.228 | 1 | 0.133 |
| **ΔAF** | 0.098 | 0.133 | 1 |
|  |  |  |  |
| **Uganda** | **Rsb** | **iHS** | **ΔAF** |
| **Rsb** | 1 | 0.22 | 0.08 |
| **iHS** | 0.22 | 1 | 0.116 |
| **ΔAF** | 0.08 | 0.116 | 1 |
|  |  |  |  |
| **Nigeria** | **Rsb** | **iHS** | **ΔAF** |
| **Rsb** | 1 | 0.22 | 0.077 |
| **iHS** | 0.22 | 1 | 0.122 |
| **ΔAF** | 0.077 | 0.122 | 1 |

**Supplementary Table 7:** Candidate regions from each genome-wide SNP analysis (*iHS*, *Rsb*, Δ*AF* and *meta-SS*) performed on the East African cattle populations from Uganda (UGN) with the combined reference populations (Holstein-Friesian, Jersey, N’Dama, Muturu, Nelore and Gir).

| **iHS** | **Rsb** | **∆AF** | **meta-SS** |
| --- | --- | --- | --- |
| BTA 1:67180060-67313350 | BTA 3:75775876-76364422 | BTA 13:48796718-48911554 | BTA 1:58355682-58612631 |
| BTA 3:120559807-121238836 | BTA 3:120604441-121247679 | BTA 13:49433476-49762965 | BTA 1:65792291-66240276 |
| BTA 5:47121805-47437350 | BTA 5:43829015-44574214 | BTA 13:50524278-50742202 | BTA 1:66748211-67328094 |
| BTA 6:4964608-5141190 | BTA 5:48149086-49174693 |  | BTA 1:149241884-149960460 |
| BTA 13:40360159-41102866 | BTA 5:60390408-60627450 |  | BTA 2:70314631-71185300 |
| BTA 19:46606725-46706542 | BTA 5:62457573-62644905 |  | BTA 2:125159084-125994861 |
|  | BTA 5:66758715-66864425 |  | BTA 3:33458232-33531571 |
|  | BTA 6:4837150-4876731 |  | BTA 3:34254043-34727876 |
|  | BTA 6:40303148-40934693 |  | BTA 3:67940307-68128218 |
|  | BTA 7:32640500-33427191 |  | BTA 3:75391246-76825243 |
|  | BTA 8:45762230-46406194 |  | BTA 3:98563369-99283161 |
|  | BTA 9:70649533-70687691 |  | BTA 3:120601191-121300696 |
|  | BTA 11:62482731-62765277 |  | BTA 5:23652016-24338695 |
|  | BTA 12:25490082-25676204 |  | BTA 5:43834751-44574214 |
|  | BTA 12:26945295-27085108 |  | BTA 5:47143913-49212943 |
|  | BTA 12:35689908-35886159 |  | BTA 5:49857890-49993761 |
|  | BTA 13:31691092-31787896 |  | BTA 5:50952073-51113065 |
|  | BTA 13:39579929-41328969 |  | BTA 5:60390408-60699053 |
|  | BTA 14:28164594-28430215 |  | BTA 5:62045272-62587423 |
|  | BTA 15:44849752-45106327 |  | BTA 5:63555403-64334857 |
|  | BTA 15:48585759-48841386 |  | BTA 5:65268314-66966194 |
|  | BTA 19:2473530-3913240 |  | BTA 5:109303999-109688098 |
|  | BTA 20:3206730-3568975 |  | BTA 6:4876731-5065951 |
|  | BTA 22:29533544-30218613 |  | BTA 6:16514162-16839617 |
|  | BTA 24:61968531-62570516 |  | BTA 7:680008-814340 |
|  |  |  | BTA 7:31130800-35059248 |
|  |  |  | BTA 7:50029472-50809190 |
|  |  |  | BTA 7:61232987-61396966 |
|  |  |  | BTA 7:62551178-62782874 |
|  |  |  | BTA 7:100845937-102014448 |
|  |  |  | BTA 8:23344221-23663852 |
|  |  |  | BTA 8:45755851-46530833 |
|  |  |  | BTA 8:60315332-60591587 |
|  |  |  | BTA 8:65373897-65634601 |
|  |  |  | BTA 9:69198185-69406467 |
|  |  |  | BTA 9:72738853-74253900 |
|  |  |  | BTA 9:75053662-76853587 |
|  |  |  | BTA 9:88390838-88469911 |
|  |  |  | BTA 9:94121197-94242831 |
|  |  |  | BTA 10:25708993-25852549 |
|  |  |  | BTA 10:76573284-77211431 |
|  |  |  | BTA 10:80515703-80796559 |
|  |  |  | BTA 11:6784799-6924499 |
|  |  |  | BTA 11:37968468-39743107 |
|  |  |  | BTA 11:61868285-62784490 |
|  |  |  | BTA 11:71387248-72221099 |
|  |  |  | BTA 11:94562083-95050103 |
|  |  |  | BTA 12:21086969-21306618 |
|  |  |  | BTA 12:24813715-26180618 |
|  |  |  | BTA 12:28949354-29572256 |
|  |  |  | BTA 12:35689908-36746504 |
|  |  |  | BTA 12:90591899-90828989 |
|  |  |  | BTA 13:18130223-18421481 |
|  |  |  | BTA 13:31639422-32688842 |
|  |  |  | BTA 13:39579929-41363364 |
|  |  |  | BTA 13:47532424-49197290 |
|  |  |  | BTA 13:50490155-50837529 |
|  |  |  | BTA 13:51339718-51522874 |
|  |  |  | BTA 13:58270096-58599491 |
|  |  |  | BTA 14:27987402-28430215 |
|  |  |  | BTA 15:44843650-45012875 |
|  |  |  | BTA 15:48555721-48841386 |
|  |  |  | BTA 15:52859008-52964463 |
|  |  |  | BTA 15:63854687-64454641 |
|  |  |  | BTA 16:25241257-25540339 |
|  |  |  | BTA 16:26807748-27160301 |
|  |  |  | BTA 16:46869577-47614377 |
|  |  |  | BTA 16:50138923-50762363 |
|  |  |  | BTA 19:1831783-3880072 |
|  |  |  | BTA 19:9515063-9780078 |
|  |  |  | BTA 19:26704580-27154113 |
|  |  |  | BTA 19:39330233-40808559 |
|  |  |  | BTA 19:44438549-47446995 |
|  |  |  | BTA 19:50168911-50624183 |
|  |  |  | BTA 20:3206730-4121521 |
|  |  |  | BTA 20:39393926-39537515 |
|  |  |  | BTA 21:33590777-33696403 |
|  |  |  | BTA 21:60026698-60449172 |
|  |  |  | BTA 21:64366520-64391303 |
|  |  |  | BTA 21:70668775-71102609 |
|  |  |  | BTA 22:29533544-30366810 |
|  |  |  | BTA 22:31696964-32034690 |
|  |  |  | BTA 22:45231901-46400273 |
|  |  |  | BTA 23:8124702-8664162 |
|  |  |  | BTA 24:61972128-62573437 |
|  |  |  | BTA 26:39451175-39523027 |

**Supplementary Table 8:** Candidate regions from each genome-wide SNP analysis (*iHS*, *Rsb*, Δ*AF* and *meta-SS*) performed on the zebu cattle populations from Nigeria with the combined reference populations (Holstein-Friesian, Jersey, N’Dama, Muturu, Nelore and Gir).

| **iHS** | **Rsb** | **∆AF** | **meta-SS** |
| --- | --- | --- | --- |
| BTA 5:47241942-48399194 | BTA 2:572692-997590 | BTA 5:47143913-49209163 | BTA 1:33254585-33443559 |
| BTA 5:48930543-49018256 | BTA 2:70314631-71209253 | BTA 7:51259460-53502939 | BTA 1:36856368-37557262 |
| BTA 5:76084996-76200813 | BTA 5:27274794-27335812 | BTA 7:54290110-55300310 | BTA 1:43720640-43985755 |
| BTA 6:4964608-5090033 | BTA 5:47112945-49253605 | BTA 12:29227227-29572256 | BTA 1:70234565-70425655 |
|  | BTA 5:56716286-57930050 |  | BTA 1:147319412-147434939 |
|  | BTA 5:58516607-58841085 |  | BTA 1:149547998-150039543 |
|  | BTA 5:60374628-60444745 |  | BTA 1:150701102-151399165 |
|  | BTA 5:62045272-62379266 |  | BTA 2:307743-1038299 |
|  | BTA 7:773076-814340 |  | BTA 2:70296102-71292148 |
|  | BTA 7:54054385-54422398 |  | BTA 3:14111392-14659428 |
|  | BTA 11:18685487-18979192 |  | BTA 3:75903912-76413468 |
|  | BTA 12:27816136-29615976 |  | BTA 3:84683280-85159851 |
|  | BTA 13:57490777-57902524 |  | BTA 3:98750825-99483458 |
|  | BTA 15:46020475-46333987 |  | BTA 3:101308813-102471446 |
|  | BTA 18:13404310-13925544 |  | BTA 4:49962548-50223466 |
|  | BTA 18:15396378-15517277 |  | BTA 4:53809476-54112087 |
|  | BTA 19:2506362-2748282 |  | BTA 4:55085910-57531310 |
|  | BTA 19:51832293-51964299 |  | BTA 4:63669888-63915653 |
|  | BTA 20:11164837-11338515 |  | BTA 4:66698519-67082312 |
|  | BTA 22:44480638-44553450 |  | BTA 5:15612090-16031807 |
|  | BTA 24:43936586-44060913 |  | BTA 5:17855007-18400249 |
|  | BTA 26:45869170-45878388 |  | BTA 5:19585877-20210838 |
|  |  |  | BTA 5:43230619-44574214 |
|  |  |  | BTA 5:46938597-49993761 |
|  |  |  | BTA 5:56006965-57930050 |
|  |  |  | BTA 5:58604207-58874619 |
|  |  |  | BTA 5:60250896-60610616 |
|  |  |  | BTA 5:62146394-63255485 |
|  |  |  | BTA 5:71095194-71137918 |
|  |  |  | BTA 5:72195598-72290257 |
|  |  |  | BTA 5:77640688-78095336 |
|  |  |  | BTA 5:120826563-121179132 |
|  |  |  | BTA 6:4782814-4876731 |
|  |  |  | BTA 6:36964005-37577335 |
|  |  |  | BTA 6:48105787-49139974 |
|  |  |  | BTA 6:78353035-78490912 |
|  |  |  | BTA 6:79803715-81241492 |
|  |  |  | BTA 6:83529875-84200299 |
|  |  |  | BTA 6:95134468-95192633 |
|  |  |  | BTA 7:680008-814340 |
|  |  |  | BTA 7:20376230-20479996 |
|  |  |  | BTA 7:32640500-33093884 |
|  |  |  | BTA 7:50029472-50670070 |
|  |  |  | BTA 7:51252285-52178639 |
|  |  |  | BTA 7:52865974-54497796 |
|  |  |  | BTA 7:56112647-56311087 |
|  |  |  | BTA 7:62415406-62941787 |
|  |  |  | BTA 8:21615512-21733252 |
|  |  |  | BTA 8:45755851-46131855 |
|  |  |  | BTA 8:53262721-53965241 |
|  |  |  | BTA 8:54724271-55231867 |
|  |  |  | BTA 8:58080010-58601842 |
|  |  |  | BTA 8:60004919-60608370 |
|  |  |  | BTA 8:96337832-96616451 |
|  |  |  | BTA 10:20524518-20813721 |
|  |  |  | BTA 10:26735446-27036997 |
|  |  |  | BTA 10:57626493-57815351 |
|  |  |  | BTA 10:93813158-93886902 |
|  |  |  | BTA 10:101246708-101611132 |
|  |  |  | BTA 10:103171282-103846097 |
|  |  |  | BTA 11:18497556-19272280 |
|  |  |  | BTA 11:47991402-49439232 |
|  |  |  | BTA 11:62343547-62919865 |
|  |  |  | BTA 12:27998214-29572256 |
|  |  |  | BTA 12:89754107-91041467 |
|  |  |  | BTA 13:18132557-18320265 |
|  |  |  | BTA 13:55428804-55616661 |
|  |  |  | BTA 13:56893876-58090472 |
|  |  |  | BTA 14:13392060-14313552 |
|  |  |  | BTA 14:30997027-31287089 |
|  |  |  | BTA 14:49149502-49374173 |
|  |  |  | BTA 15:41920165-42568640 |
|  |  |  | BTA 15:63026020-63383174 |
|  |  |  | BTA 16:25389029-25889912 |
|  |  |  | BTA 16:50138923-50936489 |
|  |  |  | BTA 18:13878200-14454853 |
|  |  |  | BTA 19:2555043-2765065 |
|  |  |  | BTA 19:27004483-27435200 |
|  |  |  | BTA 19:34613439-35140272 |
|  |  |  | BTA 19:44788419-44924467 |
|  |  |  | BTA 19:46580102-46673984 |
|  |  |  | BTA 20:3435820-3704975 |
|  |  |  | BTA 20:9913289-11170115 |
|  |  |  | BTA 20:12988915-13274192 |
|  |  |  | BTA 20:70125037-71797830 |
|  |  |  | BTA 21:33590777-33696403 |
|  |  |  | BTA 21:42271864-42565638 |
|  |  |  | BTA 21:67360105-67771364 |
|  |  |  | BTA 21:68524346-69408187 |
|  |  |  | BTA 21:70885995-71196532 |
|  |  |  | BTA 22:43706894-46126149 |
|  |  |  | BTA 24:42979783-44160038 |
|  |  |  | BTA 24:53024005-53605996 |
|  |  |  | BTA 24:61259888-62530799 |
|  |  |  | BTA 25:42193923-42561781 |
|  |  |  | BTA 26:21612594-22894146 |
|  |  |  | BTA 26:45869170-45929906 |

**Supplementary Table S9 is published separately - see the supplementary material files**

**Supplementary Table 10:** Gene desert candidate regions identified by genome-wide SNP and *Hp* analyses.

| **KEASZ** | | **East African regions** | |
| --- | --- | --- | --- |
| **Genome-wide SNP analyses** | ***Hp* analysis** | **Genome-wide SNP analyses** | ***Hp* analysis** |
| BTA 1:54,859,494-55,507,566 | BTA 1:11,070,001-11,251,624 | BTA 13:50,616,630-50,837,529 | BTA 2:70,570,001-70,811,366 |
| BTA 3:76,084,701-76,781,970 | BTA 1:54,880,001-55,141,728 | BTA 21:60,026,698-60,449,172 | BTA 9:72,890,001-72,991,383 |
| BTA 9:87,580,569-87,599,592 | BTA 1:55,150,001-55,253,859 |  | BTA 11:39,240,001-39,530,799 |
| BTA 13:50,616,630-50,837,529 | BTA 2:70,570,001-70,811,366 |  | BTA 11:39,550,001-39,683,044 |
| BTA 19:2,568,979-2,765,065 | BTA 5:78,060,001-78,184,292 |  | BTA 13:49,590,001-49,844,283 |
| BTA 21:60,026,698-60,449,172 | BTA 5:89,980,001-90,101,800 | **East and West African regions** | |
| BTA 13:48,796,718-48,911,554 | BTA 5:90,160,001-90,264,128 | **Genome-wide SNP analyses** | ***Hp* analysis** |
|  | BTA 6:51,030,001-51,137,831 |  | BTA 2:70,570,001-70,811,366 |
|  | BTA 6:52,270,001-52,381,282 |  |  |
|  | BTA 6:52,810,001-53,302,557 |  |  |
|  | BTA 6:53,400,001-53,537,934 |  |  |
|  | BTA 9:57,340,001-57,441,269 |  |  |
|  | BTA 9:71,980,001-72,171,785 |  |  |
|  | BTA 9:72,890,001-72,991,383 |  |  |
|  | BTA 9:90,910,001-91,013,212 |  |  |
|  | BTA 11:39,240,001-39,530,799 |  |  |
|  | BTA 11:39,550,001-39,683,044 |  |  |
|  | BTA 12:70,760,001-71,243,560 |  |  |
|  | BTA 12:74,990,001-75,109,791 |  |  |
|  | BTA 12:82,290,001-82,473,041 |  |  |
|  | BTA 13:49,590,001-49,844,283 |  |  |
|  | BTA 13:82,010,001-82,111,606 |  |  |
|  | BTA 14:46,100,001-46,252,557 |  |  |
|  | BTA 16:38,780,001-38,881,005 |  |  |
|  | BTA 16:41,730,001-41,885,996 |  |  |
|  | BTA 17:63,070,001-63,185,472 |  |  |
|  | BTA 18:37,400,001-37,541,995 |  |  |
|  | BTA 20:49,370,001-49,599,936 |  |  |
|  | BTA 21:40,100,001-40,201,502 |  |  |
|  | BTA 23:18,980,001-19,091,451 |  |  |
|  | BTA 24:4,660,001-4,771,543 |  |  |
|  | BTA 29:34,280,001-34,380,825 |  |  |

**Supplementary Table 11**: Genome coordinates (UMD3.1), size and mean ZHp value of candidate sweep regions in the KEASZ autosomes identified by the *Hp* sequence analysis.

| **BTA** | **start** | **stop** | **Other studies*** | **size (bp)** | mean ZHp |
| --- | --- | --- | --- | --- | --- |
| 1 | 11,070,001 | 11,251,624 |  | 181,623 | -4.21 |
| 1 | 14,910,001 | 15,065,189 |  | 155,188 | -4.54 |
| 1 | 42,100,001 | 42,221,164 |  | 121,163 | -4.20 |
| 1 | 42,520,001 | 42,621,530 |  | 101,529 | -4.10 |
| 1 | 50,440,001 | 50,622,427 |  | 182,426 | -5.81 |
| 1 | 54,880,001 | 55,141,728 |  | 261,727 | -6.56 |
| 1 | 55,150,001 | 55,253,859 |  | 103,858 | -4.12 |
| 1 | 56,220,001 | 56,392,319 |  | 172,318 | -4.53 |
| 1 | 66,910,001 | 67,064,443 |  | 154,442 | -4.58 |
| 1 | 67,310,001 | 67,710,526 |  | 400,525 | -6.98 |
| 1 | 71,180,001 | 71,283,103 |  | 103,102 | -4.11 |
| 1 | 80,510,001 | 80,660,409 |  | 150,408 | -4.37 |
| 1 | 127,270,001 | 127,400,871 |  | 130,870 | -4.71 |
| 2 | 70,570,001 | 70,811,366 | Liao *et al*., 2013; Kemper *et al*., 2014; Gautier *et al*., 2009 | 241,365 | -6.68 |
| 2 | 70,990,001 | 71,191,313 | Liao *et al*., 2013; Kemper *et al*., 2014; Gautier *et al*., 2009 | 201,312 | -5.27 |
| 2 | 106,240,001 | 106,412,107 |  | 172,106 | -5.22 |
| 2 | 111,340,001 | 111,461,100 |  | 121,099 | -4.46 |
| 2 | 125,300,001 | 125,620,820 | Gautier *et al*., 2009 | 320,819 | -8.12 |
| 2 | 125,640,001 | 126,083,262 | Gautier *et al*., 2009 | 443,261 | -7.66 |
| 3 | 57,450,001 | 57,872,320 | Kemper *et al*., 2014 | 422,319 | -7.01 |
| 3 | 65,990,001 | 66,121,297 |  | 131,296 | -4.30 |
| 4 | 64,060,001 | 64,231,634 |  | 171,633 | -4.91 |
| 4 | 75,050,001 | 75,152,011 |  | 102,010 | -4.02 |
| 5 | 24,980,001 | 25,100,643 |  | 120,642 | -4.54 |
| 5 | 47,540,001 | 48,083,606 | Liao *et al*., 2013; Kemper *et al*., 2014; Xu *et al*., 2014, Perez O'Brien *et al*., 2014 | 543,605 | -8.12 |
| 5 | 48,610,001 | 49,021,113 | Liao *et al*., 2013; Kemper *et al*., 2014; Xu *et al*., 2014, Perez O'Brien *et al*., 2014 | 411,112 | -5.01 |
| 5 | 49,120,001 | 49,241,076 | Liao *et al*., 2013; Kemper *et al*., 2014; Perez O'Brien *et al*., 2014 | 121,075 | -4.47 |
| 5 | 59,510,001 | 59,637,265 | Liao *et al*., 2013; Kemper *et al*., 2014; Gautier *et al*., 2009; Chan *et al*., 2010 | 127,264 | -4.59 |
| 5 | 60,610,001 | 60,721,361 | Liao *et al*., 2013; Kemper *et al*., 2014; Flori *et al*., 2014; Gautier *et al*., 2009; Gautier and Navas, 2011; Chan *et al*, 2010 | 111,360 | -4.33 |
| 5 | 66,500,001 | 67,010,316 | Chan *et al*., 2010 | 510,315 | -6.20 |
| 5 | 68,800,001 | 68,992,941 |  | 192,940 | -5.32 |
| 5 | 78,060,001 | 78,184,292 | Larkin *et al*., 2012 | 124,291 | -4.43 |
| 5 | 89,610,001 | 89,721,081 |  | 111,080 | -4.31 |
| 5 | 89,980,001 | 90,101,800 |  | 121,799 | -4.22 |
| 5 | 90,160,001 | 90,264,128 |  | 104,127 | -4.02 |
| 5 | 114,370,001 | 114,544,924 | Liao *et al*., 2013; Perez O'Brien *et al*., 2014 | 174,923 | -4.97 |
| 5 | 120,800,001 | 121,199,019 | Liao *et al*., 2013 | 399,018 | -8.21 |
| 6 | 5,470,001 | 5,842,010 |  | 372,009 | -4.70 |
| 6 | 5,930,001 | 6,706,107 |  | 776,106 | -7.02 |
| 6 | 12,760,001 | 12,881,667 |  | 121,666 | -4.27 |
| 6 | 51,030,001 | 51,137,831 |  | 107,830 | -4.15 |
| 6 | 52,270,001 | 52,381,282 |  | 111,281 | -4.30 |
| 6 | 52,810,001 | 53,302,557 |  | 492,556 | -10.34 |
| 6 | 53,400,001 | 53,537,934 |  | 137,933 | -6.01 |
| 6 | 60,120,001 | 60,321,420 |  | 201,419 | -6.34 |
| 6 | 60,480,001 | 60,622,934 |  | 142,933 | -4.30 |
| 6 | 81,600,001 | 81,925,350 | Perez O'Brien *et al*., 2014 | 325,349 | -5.70 |
| 6 | 94,070,001 | 94,177,765 |  | 107,764 | -4.01 |
| 6 | 99,890,001 | 100,008,555 |  | 118,554 | -4.17 |
| 7 | 440,001 | 561,436 |  | 121,435 | -4.34 |
| 7 | 22,910,001 | 23,012,434 |  | 102,433 | -4.02 |
| 7 | 31,740,001 | 31,897,059 | Flori *et al*., 2014 | 157,058 | -4.54 |
| 7 | 33,100,001 | 33,293,306 |  | 193,305 | -4.43 |
| 7 | 44,180,001 | 44,474,576 | Liao *et al*., 2013; Kemper *et al*., 2014 | 294,575 | -13.65 |
| 7 | 51,360,001 | 53,362,761 | Liao *et al*., 2013; Qanbari *et al*., 2014; Porto Neto *et al*., 2013; Gautier *et al*., 2009 | 2,002,760 | -10.79 |
| 7 | 53,720,001 | 54,521,446 | Gautier *et al*., 2009; Perez O'Brien *et al*., 2014; Gautier and Navas, 2011 | 801,445 | -6.95 |
| 7 | 54,550,001 | 54,831,282 | Gautier *et al*., 2009; Gautier and Navas, 2011 | 281,281 | -5.70 |
| 7 | 89,130,001 | 89,324,444 |  | 194,443 | -10.42 |
| 7 | 93,190,001 | 93,311,412 |  | 121,411 | -4.11 |
| 7 | 93,400,001 | 93,502,562 |  | 102,561 | -4.12 |
| 8 | 59,900,001 | 60,020,644 |  | 120,643 | -4.15 |
| 9 | 57,340,001 | 57,441,269 |  | 101,268 | -4.21 |
| 9 | 57,570,001 | 57,761,240 |  | 191,239 | -4.48 |
| 9 | 71,170,001 | 71,419,135 |  | 249,134 | -5.98 |
| 9 | 71,980,001 | 72,171,785 |  | 191,784 | -6.20 |
| 9 | 72,890,001 | 72,991,383 |  | 101,382 | -4.06 |
| 9 | 73,890,001 | 74,081,863 |  | 191,862 | -5.45 |
| 9 | 76,600,001 | 76,876,188 |  | 276,187 | -6.12 |
| 9 | 90,910,001 | 91,013,212 | Larkin *et al*., 2012 | 103,211 | -4.55 |
| 10 | 17,100,001 | 17,251,035 |  | 151,034 | -4.25 |
| 10 | 59,110,001 | 59,210,489 | Liao *et al*., 2014 | 100,488 | -4.12 |
| 10 | 76,650,001 | 76,840,921 |  | 190,920 | -5.41 |
| 11 | 7,110,001 | 7,261,196 |  | 151,195 | -5.24 |
| 11 | 37,550,001 | 37,720,588 | Kemper *et al*., 2014; Gautier *et al*., 2009 | 170,587 | -4.57 |
| 11 | 39,240,001 | 39,530,799 | Kemper *et al*., 2014; Gautier *et al*., 2009 | 290,798 | -5.93 |
| 11 | 39,550,001 | 39,683,044 | Kemper *et al*., 2014; Gautier *et al*., 2009 | 133,043 | -4.38 |
| 11 | 51,480,001 | 51,661,703 |  | 181,702 | -4.30 |
| 11 | 62,810,001 | 62,971,604 |  | 161,603 | -4.89 |
| 11 | 64,380,001 | 64,590,513 | Gautier and Navas, 2011; Flori *et al*., 2014 | 210,512 | -4.69 |
| 11 | 75,230,001 | 75,441,012 |  | 211,011 | -6.40 |
| 11 | 107,180,001 | 107,323,262 |  | 143,261 | -12.65 |
| 12 | 20,870,001 | 21,021,506 | Gautier *et al*., 2009 | 151,505 | -5.64 |
| 12 | 21,130,001 | 21,320,859 | Gautier *et al*., 2009 | 190,858 | -4.63 |
| 12 | 29,110,001 | 29,438,417 | Liao *et al*., 2013; Porto Neto *et al*., 2013; Flori *et al*., 2014; Gautier *et al*., 2009; Gautier and Navas, 2011 | 328,416 | -5.83 |
| 12 | 70,760,001 | 71,243,560 |  | 483,559 | -6.58 |
| 12 | 72,510,001 | 72,644,371 |  | 134,370 | -4.40 |
| 12 | 74,730,001 | 74,973,069 |  | 243,068 | -7.40 |
| 12 | 74,990,001 | 75,109,791 |  | 119,790 | -4.17 |
| 12 | 82,290,001 | 82,473,041 |  | 183,040 | -4.69 |
| 13 | 350,001 | 497,233 |  | 147,232 | -5.50 |
| 13 | 5,280,001 | 5,574,912 | Liao *et al*., 2013 | 294,911 | -10.45 |
| 13 | 17,480,001 | 17,590,581 |  | 110,580 | -4.37 |
| 13 | 24,480,001 | 24,623,450 | Gautier and Navas, 2011 | 143,449 | -4.71 |
| 13 | 47,980,001 | 48,164,495 |  | 184,494 | -4.99 |
| 13 | 48,330,001 | 48,460,640 |  | 130,639 | -4.96 |
| 13 | 48,650,001 | 49,056,444 | Porto Neto *et al*., 2013 | 406,443 | -10.07 |
| 13 | 49,340,001 | 49,551,378 | Porto Neto *et al*., 2013 | 211,377 | -5.80 |
| 13 | 49,590,001 | 49,844,283 | Porto Neto *et al*., 2013 | 254,282 | -6.78 |
| 13 | 50,240,001 | 50,852,056 |  | 612,055 | -7.32 |
| 13 | 51,310,001 | 51,521,330 |  | 211,329 | -4.73 |
| 13 | 51,600,001 | 51,800,737 | Liao *et al*., 2013 | 200,736 | -6.18 |
| 13 | 52,040,001 | 52,141,150 |  | 101,149 | -4.04 |
| 13 | 55,510,001 | 55,623,671 |  | 113,670 | -4.48 |
| 13 | 55,710,001 | 55,833,923 |  | 123,922 | -4.43 |
| 13 | 57,990,001 | 58,122,843 | Kemper *et al*., 2014; Flori *et al*., 2014 | 132,842 | -4.89 |
| 13 | 82,010,001 | 82,111,606 |  | 101,605 | -4.06 |
| 14 | 660,001 | 1,085,829 |  | 425,828 | -4.43 |
| 14 | 2,190,001 | 2,405,393 |  | 215,392 | -6.33 |
| 14 | 41,390,001 | 41,563,443 | Flori *et al*., 2014 | 173,442 | -5.30 |
| 14 | 46,100,001 | 46,252,557 | Gautier *et al*., 2009 | 152,556 | -4.51 |
| 14 | 83,120,001 | 83,233,061 |  | 113,060 | -4.56 |
| 14 | 83,380,001 | 83,483,495 |  | 103,494 | -4.10 |
| 15 | 59,500,001 | 59,741,560 |  | 241,559 | -5.22 |
| 15 | 85,090,001 | 85,272,412 |  | 182,411 | -5.36 |
| 16 | 33,120,001 | 33,222,179 |  | 102,178 | -4.24 |
| 16 | 38,780,001 | 38,881,005 | Kemper *et al*., 2014; Chan *et al*., 2010 | 101,004 | -4.92 |
| 16 | 41,730,001 | 41,885,996 | Kemper *et al*., 2014; Chan *et al*., 2010 | 155,995 | -4.24 |
| 16 | 44,660,001 | 44,861,757 | Liao *et al*., 2013; Kemper *et al*., 2014; Chan *et al*., 2010 | 201,756 | -7.59 |
| 17 | 35,600,001 | 35,745,362 |  | 145,361 | -5.19 |
| 17 | 35,760,001 | 36,031,948 | Liao *et al*., 2013 | 271,947 | -7.68 |
| 17 | 49,010,001 | 49,134,618 | Perez O'Brien *et al*., 2014 | 124,617 | -4.12 |
| 17 | 50,750,001 | 51,039,324 |  | 289,323 | -7.79 |
| 17 | 51,130,001 | 51,676,138 |  | 546,137 | -6.30 |
| 17 | 51,840,001 | 52,101,083 |  | 261,082 | -6.02 |
| 17 | 63,070,001 | 63,185,472 |  | 115,471 | -4.46 |
| 18 | 23,180,001 | 23,634,981 |  | 454,980 | -8.03 |
| 18 | 36,500,001 | 36,700,717 |  | 200,716 | -4.64 |
| 18 | 37,010,001 | 37,390,731 |  | 380,730 | -4.40 |
| 18 | 37,400,001 | 37,541,995 |  | 141,994 | -4.43 |
| 18 | 50,540,001 | 50,663,542 | Gautier *et al*., 2009 | 123,541 | -4.66 |
| 19 | 9,500,001 | 9,631,079 |  | 131,078 | -4.72 |
| 19 | 12,460,001 | 12,671,997 |  | 211,996 | -4.80 |
| 19 | 26,890,001 | 27,154,002 | Gautier *et al*., 2009 | 264,001 | -7.60 |
| 19 | 27,490,001 | 27,674,039 | Gautier *et al*., 2009; Liao *et al*., 2013 | 184,038 | -4.46 |
| 19 | 27,970,001 | 28,092,385 | Gautier *et al*., 2009; Liao *et al*., 2013 | 122,384 | -4.39 |
| 19 | 39,270,001 | 39,422,844 |  | 152,843 | -4.42 |
| 19 | 40,490,001 | 40,714,976 |  | 224,975 | -4.60 |
| 19 | 40,960,001 | 41,450,870 |  | 490,869 | -5.98 |
| 19 | 42,890,001 | 43,122,753 | Chan *et al*., 2010 | 232,752 | -6.09 |
| 19 | 43,140,001 | 43,341,262 | Chan *et al*., 2010 | 201,261 | -6.53 |
| 20 | 2,790,001 | 2,895,925 | Kemper *et al*., 2014 | 105,924 | -4.02 |
| 20 | 48,720,001 | 49,031,220 |  | 311,219 | -5.35 |
| 20 | 49,370,001 | 49,599,936 |  | 229,935 | -6.83 |
| 21 | 1,830,001 | 1,955,744 |  | 125,743 | -4.31 |
| 21 | 40,100,001 | 40,201,502 |  | 101,501 | -4.09 |
| 22 | 2,890,001 | 3,057,549 |  | 167,548 | -5.84 |
| 22 | 30,030,001 | 30,260,687 |  | 230,686 | -6.05 |
| 22 | 39,720,001 | 40,077,687 | Liao *et al*., 2013 | 357,686 | -12.27 |
| 22 | 41,820,001 | 41,970,661 | Gautier *et al*., 2009; Chan *et al*., 2010 | 150,660 | -5.09 |
| 22 | 45,220,001 | 45,370,457 | Gautier *et al*., 2009; Chan *et al*., 2010; Flori *et al*., 2014 | 150,456 | -4.17 |
| 23 | 350,001 | 644,988 | Perez O'Brien *et al*., 2014 | 294,987 | -4.55 |
| 23 | 18,520,001 | 18,691,851 |  | 171,850 | -4.36 |
| 23 | 18,790,001 | 18,890,398 |  | 100,397 | -4.28 |
| 23 | 18,980,001 | 19,091,451 |  | 111,450 | -4.12 |
| 23 | 24,460,001 | 24,580,718 |  | 120,717 | -4.85 |
| 24 | 4,660,001 | 4,771,543 |  | 111,542 | -4.24 |
| 25 | 39,250,001 | 39,406,618 |  | 156,617 | -4.10 |
| 26 | 16,070,001 | 16,291,986 |  | 221,985 | -5.72 |
| 26 | 21,070,001 | 21,194,173 |  | 124,172 | -4.24 |
| 26 | 39,260,001 | 39,481,524 |  | 221,523 | -4.35 |
| 27 | 26,310,001 | 26,453,084 |  | 143,083 | -4.85 |
| 29 | 90,001 | 300,385 |  | 210,384 | -5.95 |
| 29 | 17,780,001 | 17,880,857 |  | 100,856 | -4.06 |
| 29 | 34,280,001 | 34,380,825 |  | 100,824 | -4.07 |
| 29 | 45,320,001 | 45,462,334 |  | 142,333 | -4.69 |

**Supplementary Table S12 is published separately - see the supplementary material files**

**Supplementary Table S13 is published separately - see the supplementary material files**

**Supplementary Table S14 is published separately - see the supplementary material files**

**Supplementary Table S15 is published separately - see the supplementary material files**

**Supplementary Table S16 is published separately - see the supplementary material files**

**Supplementary Table 17:** : Candidate regions identified in the genome of KEASZ using Illumina BovineSNP50 BeadChip v.1 (Bahbahabi *et al.* 2015) and their overlapping candidate regions in the genome of KEASZ identified in the current study.

| **Bahbahani *et al.* (2015)** | | | **Current study** | |
| --- | --- | --- | --- | --- |
| **BTA** | **Chromosomal Position** | **Identifying Statistic** | **Genome-wide SNP analysis** | **Pooled heterozygosity (*Hp*) analysis** |
| 2 | 125,585,810 – 126,058,677 | *F_ST_* | 125,159,084 – 125,994,861 | 125,640,001 – 126,083,262 |
| 3 | 101,942,771 | *Rsb* | _ | _ |
| 4 | 47,195,467 – 47,539,595 | *F_ST_* | _ | _ |
| 4 | 51,927,595 – 52,308,430 | *F_ST_* | _ | _ |
| 5 | 57,977,594 | *Rsb* | 56,651,062 – 57,515,653 | _ |
| 5 | 60,556,520 | *Rsb* | _ | 60,610,001 – 60,721,361 |
| 5 | 76,286,670 | *iHS* | _ | _ |
| 7 | 52,224,595 – 52,720,797 | *F_ST_* | 52,183,599 – 53,001,042 | 51,360,001 – 53,362,761 |
| 11 | 62,629,106 | *Rsb* | 61,877,437 – 62,548,419 | 62,810,001 – 62,971,604 |
| 12 | 27,181,474 | *Rsb* | 27,050,192 – 29,151,436 | _ |
| 12 | 29,217,254 | *Rsb* | 27,050,192 – 29,151,436 | 29,110,001 – 29,438,417 |
| 12 | 35,740,174 | *Rsb* | 35,445,176 – 36,965,854 | _ |
| 13 | 46,433,697 – 46,723,493 | *F_ST_* | _ | _ |
| 13 | 57,848,276 – 58,207,174 | *F_ST_* | 58,273,562 – 58,599,491 | 57,990,001 – 58,122,843 |
| 14 | 24,482,969 – 25,254,540 | *F_ST_* | _ | _ |
| 19 | 27,369,763 – 27,763,447 | *F_ST_* | 26,909,816 – 27,143,239 | 27,490,001 – 27,674,039 |
| 19 | 42,696,815 | *Rsb* | 43,023,638 – 43,692,285 | 42,890,001 – 43,122,753 |
| 22 | 2,314,019 – 2,788,566 | *F_ST_* | _ | 2,890,001 – 30,5754,9 |
| 23 | 28,281,915 | *iHS* | _ | _ |
| 24 | 4,118,163 – 4,474,760 | *F_ST_* | _ | 4,660,001 – 4,771,543 |
| 29 | 1,898,171 | *iHS* | _ | _ |
| X | 8,582,093 – 9,248,137 | *F_ST_* | _ | _ |
| X | 39,942,044 – 42,024,368 | *F_ST_* | _ | _ |
| X | 84,566,018 – 85,993,719 | *F_ST_* | 86,750,029 – 87,174,478 | _ |

**Supplementary Table S18 is published separately - see he supplementary material files**

**Supplementary Table S19 is published separately - see he supplementary material files**
